# Phyto-Nanocatalysts: Green Synthesis, Characterization, and Applications

**DOI:** 10.3390/molecules24193418

**Published:** 2019-09-20

**Authors:** Radu Claudiu Fierascu, Alina Ortan, Sorin Marius Avramescu, Irina Fierascu

**Affiliations:** 1 National Institute for Research & Development in Chemistry and Petrochemistry–ICECHIM Bucharest, 060021 Bucharest, Romania; radu_claudiu_fierascu@yahoo.com; 2 University of Agronomic Sciences and Veterinary Medicine of Bucharest, 011464 Bucharest, Romania; 3 Research Center for Environmental Protection and Waste Management, University of Bucharest, 050107 Bucharest, Romania; sorin_avramescu@yahoo.com

**Keywords:** phyto-nanocatalysts, plant extracts, environmental applications, mono- and bi-metallic catalysts, metallic oxides nanoparticles, complex catalysts

## Abstract

Catalysis represents the cornerstone of chemistry, since catalytic processes are ubiquitous in almost all chemical processes developed for obtaining consumer goods. Nanocatalysis represents nowadays an innovative approach to obtain better properties for the catalysts: stable activity, good selectivity, easy to recover, and the possibility to be reused. Over the last few years, for the obtaining of new catalysts, classical methods—based on potential hazardous reagents—have been replaced with new methods emerged by replacing those reagents with plant extracts obtained in different conditions. Due to being diversified in morphology and chemical composition, these materials have different properties and applications, representing a promising area of research. In this context, the present review focuses on the metallic nanocatalysts’ importance, different methods of synthesis with emphasis to the natural compounds used as support, characterization techniques, parameters involved in tailoring the composition, size and shape of nanoparticles and applications in catalysis. This review presents some examples of green nanocatalysts, grouped considering their nature (mono- and bi-metallic nanoparticles, metallic oxides, sulfides, chlorides, and other complex catalysts).

## 1. Phyto-Nanocatalysts (PNC)–Next Generation of Catalytic Materials

Catalysis represents the cornerstone of chemistry since catalytic processes are ubiquitous in almost all chemical processes developed for obtaining consumer goods. Started about 200 years ago, as an empirical scientific field and no more than an unusual aspect of chemistry, catalysis gains more and more attention due to his importance in obtaining new compounds. More than 90% of industrial products (pharmaceutical, fibers, fuels, detergents, polymers) result through several steps which need an appropriate catalyst. The role of catalyst in the process development consists not only to accelerate the chemical reaction, but also to improve the whole process, by reducing energy consumption, the formation of undesired products, and production increase. Our society, as we know, does not exist in the absence of catalysis.

Over the last few decades, catalysis has been successfully applied in developing methods for environmental protection, with notable examples being the three ways catalyst for purification of exhaust gases [1] and water treatment using catalysts in the frame of advanced oxidation processes [2,3,4]. Catalysis evolution encompasses two main categories: homogeneous type, when reactants and catalysts are in the same phase, and heterogeneous type, when reactant and catalysts are in different phases. Heterogeneous catalysis represents the real breakthrough in this field due to the numerous advantages; therefore, a large number of scientific papers and patents were focused on developing new catalytic processes. However, the unchallenged performances of heterogeneous catalysis come with some drawbacks. Catalyst comes in the form of porous solids with internal channels and pores, in different shapes and sizes, in order to ensure a large number of active centers [5]. This morphology leads to some difficulties in modeling and experimental set-up of catalytic reactors due to diffusion phenomena and fluid transport processes. Therefore, a new approach is required to surpass the disadvantages of both types of catalysis and to combine their impressive advantages. Nowadays, nanocatalysis represents a critical way to combine all features of previous approaches: accessibility to catalytic sites, stable activity, and selectivity, easy to recover, and with the possibility to be reused [6,7]. Nanotechnology represents a significant step forward in many domains, not only in catalysis. From bulk to nanoparticles smaller than 100 nm, the properties of materials are considerably changing; hence, there is the possibility of a wide variation of applications, especially in catalytic processes. For example, cobalt oxide is less active for the oxidation of CO to CO_2_ in bulk state even at a high temperature, but very active at room temperature in nano form. Additionally, the gold nanoparticles are very active in an extensive range of reactions [8], however, while they are in bulk state, they are inactive. Utilization of nanoparticles brings many improvements in processes where they are involved: cost-efficient, safer catalyst, reduced chemical waste, lower energy consumption, reduced global warming [9]. Even in the range of nanometric dimensions, the nano stable properties are shifted to a large extent. The color of suspension of CdSe nanoparticles is shifted from blue to red when particle size change from 2 to 8 nm [10,11]. Nanomaterials with catalytic properties are classified after their dimensionality [7] such us zero-dimensional (0D) (nonporousnanoparticles) [12,13], one dimension (1D) (nanorods, nanofibers) [12], two dimensional (2D) (nanosheets) [14] and three-dimensional (3D) nanomaterials, which include all types of nanoparticles in different shapes (nanorods, nanoshells, nanocages) [6]. Another essential feature of nanoparticles is an outstanding possibility of functionalization, which increases the number of applications in all technical fields not only in catalysis. The versatility of functionalized nanomaterials makes them so valuable and fit perfectly the gap between homogeneous and heterogeneous catalysis. Preparation of nanoparticles tailored in different forms and composition would be difficult without thorough investigations using modern characterization tools [9,15,16]: ultraviolet–visible (UV-Vis) spectroscopy, Fourier transform infrared spectroscopy (FTIR), X-ray diffraction (XRD), X-ray fluorescence (XRF), X-Ray photoelectron spectroscopy (XPS), etc. These are complementary techniques that assure a complete assessment of particle properties and therefore an accurate modality to assign the appropriate applications. The main advantages of using nanoparticles (NPs) in catalysis are:

- The possibility of high scale production of nanoparticles.
- Long-term stability of the nanoparticles.
- Controlled functionalization of nanoparticles can be achieved.

In order to use nanoparticles in chemical reactions, adequate preparation methods are needed. Since these materials are diversified in morphology and chemical composition, different methods were developed. All these methods fall into two main categories: top down synthesis and bottom-up synthesis [17,18]. The top-down synthesis relies on destructive methods that bring bulk materials to nano dimensions state. For this category, mechanical milling, chemical etching, and sputtering are the most common methods used. The bottom-up synthesis uses an opposite approach, generally starting from atomic or molecular state and building up to nanoparticle dimensions (1–100 nm). Spinning, template-based synthesis, plasma or flame spraying, laser pyrolysis, atomic or molecular condensation, chemical vapor deposition, sol-gel, precipitation, solvothermal processing, microemulsion, and sonochemistry are the usual methods for bottom-up approach. These methods are *state-of-the-art* in nanoparticles manufacturing and allow the preparation of a broad range of solid materials. However, this technique either requires some unique and expensive equipment with high capital costs or expensive and environmentally hazardous reagents. Preparation of nanoparticles in the bottom-up mode with or without the use of reagent is mainly based on the chemical reduction or electrochemical reduction processes. Generally, metal salts are reduced using sodium borohydride, cetyltrimethylammonium bromide, ascorbates, glucose, and citrates [19,20,21]. The nature of the reducing agent determines the size of the nanoparticles. Strong reducing agents produce bigger particles, while mild reducing agents induce formation of nanoparticles with smaller dimensions. Particle morphology control is achieved by utilizing appropriate surfactants (sodium dodecyl sulfate, Triton X, sodium formaldehyde sulfoxylate) and stabilizers (polyvinyl pyrrolidine (PVP), polymethylmethacrylate (PMMA), polyethylene glycol (PEG) and polymethyl acrylic acid (PMAA)). Sonochemical reduction of transitional metals occurs through acoustic cavitation and particle growth in a sonicated liquid medium. Improvement of nanotechnology field in general and nanocatalysis, in particular, require, besides the development of new catalytic systems, better preparation methods with less energy consumption and less harmful reagents. Hence, a clean, biocompatible, and eco-friendly process to synthesize nanoparticles is needed.

Over the last few years, new nanoparticle preparation methods have emerged by replacing the usual reagents with plant extracts obtained in different conditions. Plant extracts were used from centuries, as cures in folk medicine and since they have valuable properties, they are also employed in present days being a serious competitor for synthetic pharmaceuticals. The market of medicinal plants increases at a rapid pace each year and predicts for the year 2029 are that it will surpass USD 129 billion in revenue. The herbal medicine field has experienced a revival in recent years due to the growing demand among consumers for ecological oriented chemical-free treatments. The main reason for the boost in utilization of plant extracts consists of the synergic effect of all components, which belong to a broad range of classes of organic compounds, and therefore, increase the therapeutic effects. The complexity of herbal extracts is precisely the reason why these liquids can be selected as substrates for nanoparticles synthesis, since they contain simultaneously reducing, stabilizers, and surfactants agents with natural origins, and hence, eco-friendly properties.

Transition to this approach was made based on substantial evolution over the last 20 years of research strategies in the area of natural product chemistry. Advanced extraction and separation methods [22,23] (hyphenated techniques: High-performance liquid chromatography-mass spectrometry (HPLC–MS), HPLC-nuclear magnetic resonance (HPLC-NMR), HPLC–MS–NMR, HPLC–diode array detector–MS–NMR, centrifugal partition chromatography) allow researchers to improve the isolation of secondary metabolites that help to build extensive databases of phytoconstituents present in different plants. Development of chemically engineered extract, dereplication, chemogenomics, metabolomics, fingerprinting, and chemical fingerprinting bring researchers a selection base for new compounds able to participate in nanoparticles synthesis. Diversification of extraction methods [24,25], with classic (Soxhlet extraction, percolation, maceration, hydro distillation, and steam distillation) or with modern instruments such as microwave-assisted extraction, accelerated solvent extraction, ultrasound-assisted extraction, enzyme assisted extraction, pulsed electric field extraction, supercritical fluid extraction, ohmic-assisted hydro distillation, and utilization of numerous solvents with high variation of polarity open the opportunity to obtain a more tailored extracts, with the appropriate composition for obtaining nanoparticles. The main reason for developing a plant-mediated synthesis of nanocatalysts is the staggering number of plat metabolites which can be used, and removing the use of potential hazardous reagents.

For example, more than 8000 polyphenolic compounds are already identified in different plant species [24]. The most majority occur in conjugated forms with sugar residues linking with compounds like lipids, organic acids, amines, or even other phenols is very usual. Other important vegetal constituents (alkaloids, tannins, iridoids, secoiridoids, coumarins, and terpenoids), also in large numbers, can sustain an ecological path for preparing nanocatalysts.

**Figure 1:**
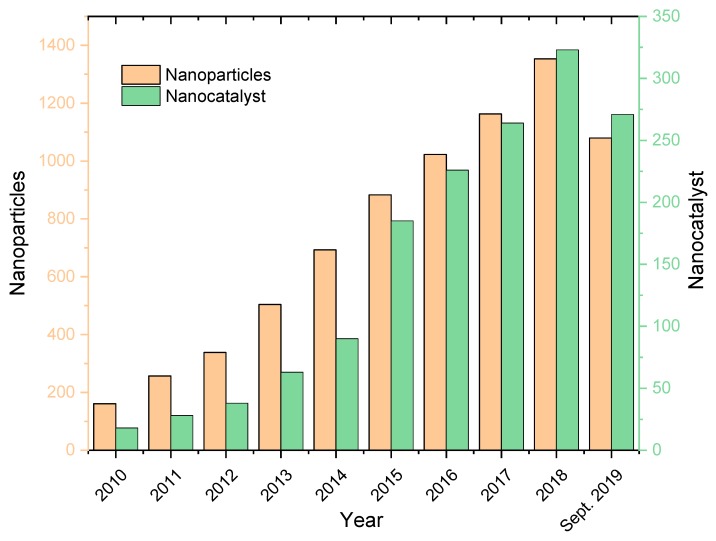
Distribution of publications per year from Scopus Database according to the presented keywords, with duplicate data and “false-positive” results removal.

The field of nanomaterials phytosynthesis in general (and nanocatalysts phytosynthesis in particular) represented the focus of an increasing number of studies in the last decades, from all-over the world, as proven by the survey performed using Scopus database (Figure 1). The search was performed over the time period 2010–September 2019, using the keywords “nanoparticle extract”, “nanoparticle phytosynthesis”, and “nanoparticle natural extract”, with “catalyst” within the obtained results; multiple keywords were used, as the term “phytosynthesis” is not adopted by all authors.

Considering these aspects, the current review focuses on the metallic nanocatalysts’ importance, different methods of synthesis with emphasis to the natural compounds used as support, characterization techniques, parameters involved in tailoring the composition, size and shape of nanoparticles, and possible applications (as reported in the literature). The review will briefly present some examples of green nanocatalysts, grouped considering their nature (mono- and bi-metallic nanoparticles, metallic oxides, sulfides, chlorides, and other complex catalysts).

## 2. Mono- and Bi-Metallic Catalysts: Phytosynthesis, Characterization, and Application

The most encountered materials, when speaking of phytosynthesized nanoparticles, are represented by the noble metal nanoparticles (especially silver and gold). Several review and research papers were published in the last years, covering the green synthesis (including phytosynthesis) of monometallic and bimetallic nanoparticles [26,27,28,29,30,31,32], with focus especially on their biological properties. The following subchapters will present some examples on the application of such nanoparticles in the area of catalysis.

### 2.1. Silver Nanoparticles (AgNP)

Phytosynthesis of silver nanoparticles (AgNP) was described by Gangula et al. [33] using the stem extract of *Breynia rhamnoides*, the reduction of the metal salts being attributed by the authors to the phenolic glycosides and reducing sugars present in the extract. The obtained nanoparticles were used for the reduction of 4-nitrophenol to 4-aminophenol (4-AP) in the presence of NaBH_4_, reaction that was found to be depend on the nanoparticle size. In 2014, Vidhu and Philip [34] obtained almost spherical silver nanoparticles, with diameters of approximately 20 nm, having excellent catalyst properties for the reduction of Methylene blue.

Silver nanoparticles were obtained using aqueous extracts of dried *Alstonia macrophylla* leaves by Borase et al. [35], having an average diameter of 70 nm. The nanoparticles proved to be efficient in the catalytic reduction of 4-nitrophenol and *p*-nitroaniline to 4-aminophenol, and p-phenylenediamine, respectively. Similar silver nanoparticles were obtained by Vidu and Phillip [36] and Nasrollahzadeh et al. [37] using aqueous extracts of *Trigonella foenum-graecum* seeds and *Euphorbia condylocarpa M. bieb* root. The nanoparticles were evaluated by the authors for the catalytic degradation of methyl orange, methylene blue and eosin Y [36] for the catalytic synthesis of N-monosubstituted ureas in water [37], with promising results.

Reduction of 4-nitrophenol to 4-aminophenol was also studied by Naraginti and Sivakumar [38] and Seralathan et al. [39] using silver nanoparticles obtained *via* aqueous *Coleus forskohlii* root, and *Salicornia brachiate* extract, while the reduction of methylene blue by phytosynthesized silver nanoparticles was studied by Ashokkumar et al. [40,41]. The differences in terms of catalytic activity observed between the two studies can be assigned to the variation in particle size.

Using phytosynthesized silver nanoparticles, reduction of 4-nitrophenol to 4-aminophenol was achieved in 2015 by Bindhu and Umadevi [42] and Joseph and Mathew [43]. Gavade et al. [44] obtained silver nanoparticles (20–30 nm) using aqueous *Ziziphus jujuba* leaf extract for the reduction of the silver salt, which was successfully used for the reduction of 4-nitrophenol and methylene blue. Degradation of methylene blue was also achieved by Ajitha et al. [45,46] using silver nanoparticles phytosynthesized by *Momordica charantia* leaf broth and *Lantana camara* leaf extract. The authors also propose a mechanism for the phytosynthesis of AgNP using *M. charantia,* assigning the main role in the silver reduction process to the flavonoid content of the extract. Several environmentally hazardous organic dyes (methylene blue, methyl orange, and methyl red) were degraded by Ahmed et al. [47] using silver nanoparticles obtained using a Ferredoxin–NADP+ reductase/ferredoxin (from spinach leaves). Two other studies published in the same year describes the catalytic application of phytosynthesized AgNP for the degradation of cellulose by cellulase [48] and for the synthesis of propargylamines [49].

Reduction of 4-nitrophenol using AgNP obtained by a greener route (involving the use of commercially available gallic acid, a phytoconstituent commonly found in natural extracts) was presented in 2016 by Park et al. [50]. In the same year, several other studies successfully applied the phytosynthesized AgNP for the degradation of dyes: direct yellow-12 [51], Acridine Orange [52], Congo red and methyl orange [53], cresyl blue [54], and methylene blue [55].

A reduction of 4-nitrophenol by AgNP was also presented in 2017 by Bello et al. [56] (reaching 95% degradation after 36 min), Arya et al. [57], Manjari et al. [58], Farhadi et al. [59], Francis et al. [60], Bonigala et al. [61], Muthu and Priya [62], Karthika et al. [63], Mohanty and Jena [64], Patil et al. [65], and Naraginti and Li [66]. Similar catalytic properties of silver nanoparticles were demonstrated for the degradation of Congo red [56,63], methylene blue and Congo red [57,58], methylene blue, methyl orange and methyl red [61], methyl orange [62], methylene blue [64,66], eosin Y (>97% degradation after 60 min) [67], Congo red [63,68], Coomassie Brilliant Blue G-250 [69], and a reduction of *p*-nitroaniline to *p*-phenylenediamine [70]. Phytosynthesized silver nanoparticles were also evaluated as catalysts for hydration of cyanamides in aqueous medium [71] and for the construction of pyrimido[1,2-b]indazole derivatives under solvent-free conditions [72].

In 2018 studies, Zaheer [73] and Arya et al. [74] presented the catalytic degradation of the carcinogenic dye 4-nitrophenol, using silver nanoparticles phytosynthesized in aqueous palm date fruit extract and aqueous *Prosopis juliflora* bark extract. The two studies noticed a 95% degradation in 8 min, correlated with the amount of AgNP used, respectively a 90% degradation in 80 min. Zaheer [73] proposed a mechanism for the phytosynthesis of the AgNP, assigning the main role in the reduction of the silver salt to the pyranoid fructose present in the fruit extract. Vijayan et al. [75,76] presented the reduction of *o*/*p*-nitroanilines, respectively, 4-nitrophenol and *o*/*p* nitroaniline catalyzed by AgNP obtained using leaf extract of *Indigofera tinctoria* andaqueous extract of *Orthosiphon aristatus* leaves, respectively. In the first study, the reaction was completed in 10 min for both *p*- and *o*-nitroaniline, while in the second case, the reactions were completed in 6 min for *o-/p-* nitroaniline and in 10 min for 4-nitrophenol. Similar observations (but with longer reaction times) were made by Francis et al. [77] for the reduction of 4-nitrophenol, 2-/4-nitroaniline, and eosin Y, using AgNP obtained by aqueous leaves extract of *Elephantopus scaber*. Reduction of 4-nitrophenol, methylene blue, methyl orange and methyl red, and, respectively, 4- nitrophenol, methylene blue and Congo red, was described by Bonigala et al. [78] and Vijayan et al. [79], using AgNP obtained by the application of *Stemona tuberosa* Lour and *Myxopyrum serratulum* A.W. Hill extracts.

Photocatalytic degradation of methylene blue was monitored by Khan et al. [80] and Sumitha et al. [81] using extracts of Longan fruit peel and *Durio zibethinus* seeds. Their results showed a 99% degradation of methylene blue in 7 min and 73.49% in 180 min, respectively. The differences between their results can most probably be attributed to the differences both in terms of morphology and average size between the obtained NPs (as presented in Table 1). Application of AgNPs for the degradation of several other dyes (Reactive Black 5, methyl orange, direct yellow-142; Coomassie Brilliant Blue; methylene blue, eosin yellowish, safranin, direct dye, reactive dyes; methyl orange, rhodamine B; Rose Bengal) was further investigated in a series of research papers, using AgNP phytosynthesized via *Convolvulus arvensis* [82], *Gardenia jasminoides* Ellis [83], *Allium cepa* [84], *Nervalia zeylanica* [85] and *Bauhinia tomentosa* Linn. [86] extracts.

An extensive study was also published by Nakkala et al. [87] on the phytosynthesis of silver nanoparticles using aqueous rhizome extract of *Acorus calamus*. The authors evaluated the obtained AgNP for *in vitro* anticancer activity, in vivo toxicity in rats, as well as for the catalytic degradation of several dyes: 4-nitrophenol, 3-nitrophenol, 2, 4, 6-trinitrophenol, picric acid, Coomassie brilliant blue, Congo red, eosin Y, rhodamine B, methylene blue, methyl red, methyl orange, cresol red, acridine orange, eriochrome black T, and phenol red. The degradation reactions were completed in 6–60 min, for all the tested dyes.

In a study published in 2019, Wang et al. [88] evaluated the catalytic properties of AgNP obtained using aqueous *Ginkgo biloba* leaves extract for the reduction of 4-nitrophenol, Congo red, methyl orange, and rhodamine B, achieving full conversion in 15–100 min. Vijayan et al. [89] applied an aqueous extract for the phytosynthesis of AgNP and evaluated the obtained materials for the catalytic degradation of methylene blue and rhodamine B. Yu et al. [90] evaluated the influence of reaction temperature on the phytosynthesis of AgNP via *Eriobotrya japonica* (Thunb.) leaves extract. Their observations were that the reaction temperature had a direct effect on the phytosynthesized nanoparticles (the higher the temperature, the larger the obtained NPs) and, as a consequence, on their catalytic properties.

Silver nanoparticles are, by far, the most encountered phytosynthesized metallic nanoparticles, mainly due to their known antimicrobial properties; the last years witnessed the increased application of the silver nanoparticles in other areas, including in catalytic applications. As is the case for the other nanoparticles, the efficiency of their final application and catalytic rates is related to their particle size and shape. The morphology of the obtained nanostructures depends on phytochemical composition of the extracts, which in turn varies depending on different extraction parameters. In perspective, for the improvement of AgNP phytosynthesis, it is necessary to optimize the synthesis process (in terms of used extract composition); another viable perspective is represented by the use of other groups of plant (less explored in comparison with common angiosperms and algae) like bryophytes and pteridophytes. These somewhat under-exploited plants contain highly oxidant agents and might be involved in producing stable nanoparticles.

### 2.2. Gold Nanoparticles (AuNP)

Although not as abundant as the papers regarding AgNP, several research articles offer numerous examples regarding the application of phytosynthesized gold nanoparticles (AuNP) in catalytic processes. In 2010, Gupta et al. [91] proposed the application of AuNP phytosynthesized using green tea for the catalytic reduction of methylene blue in the presence of stannous chloride. In 2012, Ghosh et al. applied phytosynthesized AuNP for the reduction of 4-nitrophenol [92], while in 2014, several studies present the application of phytosynthesized AuNP for the reduction of 4-nitrophenol [93,94] or methylene blue [94]. Application of phytosynthesized AuNP for the degradation of 4-nitrophenol was also described in 2016 by Borhamdin et al. [95], Ghosh et al. [96] and Lim et al. [97]; the reduction of the anthropogenic pollutant *o*-nitroaniline using AuNP was presented by Dauthal and Mukhopadhyay [98], who also proved their catalytic recyclability, while Choudhary et al. [99] presented the phytosynthesis of AuNP using *Lagerstroemia speciosa* leaf extract and their application for the reduction of different pollutants in wastewater (methylene blue, methyl orange, bromophenol blue, bromocresol green, and 4-nitrophenol). The authors also suggested a mechanism for the phytosynthesis: according to the authors, the biomolecules from the extract (such as penta-*O*-galloyl-d-glucopyranose, lagerstroemin, corosolic acid) attract negatively charged gold anions, while (through the transformation of phenolic C-OH groups to C=O keto) releasing electrons involved in the reduction of gold anions. In two 2017 studies, phytosynthesized AuNP were applied for the reduction of 4-nitrophenol [100] and methylene blue [101]. The application of AuNP for the reduction of 4-nitrophenol was also presented by Wacławek et al. [102] in their 2018 study. The authors managed to tune the shapes and sizes of the nanoparticles as a function of the pH of the synthesis solution, extract and gold salt concentration. Additionally, the comparison of the catalytic properties of different shaped AuNP (spherical and triangular) showed that the reduction of 4-nitrophenol was achieved 1.5 times faster using triangular AuNP.

Several other studies describe the application of phytosynthesized gold nanoparticles, by comparison with silver nanoparticles. The gold nanoparticles phytosynthesized by Gangula et al. [33] using *Breynia rhamnoides* extract showed superior catalytic properties for the reduction of 4-nitrophenol compared with the silver nanoparticles. A similar observation was made by Joseph and Mathew [43], who observed superior catalytic activity of gold nanoparticles phytosynthesized using aqueous *Aerva lanata* leaves extract, compared with silver nanoparticles and by Francis et al. [60] for the degradation of 4-nitrophenol, rhodamine B and methyl orange. On the contrary, Park et al. [50] noted superior catalytic activity for AgNP, compared with AuNP, both types of NPs obtained using gallic acid as a reducing agent. This can also be explained by the lower dimensions of the AgNPs, compared with AuNPs. A similar observation was made by Manjari et al. [58], who observed minor differences between the degradation capacities of AuNP and AgNP towards methylene blue and Congo red (99% for AuNP, 99.9% for AgNP) and 4-nitrophenol (95% for AuNP and 97% for AgNP), also correlated with the variation of the particle dimensions. In a 2017 study, Karthika et al. [63] found gold nanoparticles to have inferior catalytic properties for the degradation of 4-nitrophenol and Congo red compared with AgNP.

The comparative study of Vijayan et al. [75] regarding the application of AgNP and AuNP for the reduction of *o*- and *p*-nitroaniline showed inferior results for AuNP (complete degradation time 18 min), that the authors assigned to the irregular shape of AuNP (although the NPs had similar dimensions). Similar, to the irregular shapes of the AuNP obtained was assigned by Bonigala et al. [78] and Vijayan et al. [79] the inferior catalytic properties, compared with AgNP, observed for the reduction of 4-nitrophenol, methylene blue, methyl orange and methyl red, and, respectively, 4- nitrophenol, methylene blue, and Congo red. The AuNP obtained by Vijayan et al. [88] using *Bauhinia purpurea* leaf extract presented longer times (when compared with AgNP obtained in the same conditions). Again, it can be assumed that the differences in terms of catalytic activity are mainly due to the differences observed in terms of morphology.

Recently, a very interesting review paper was published by Teimouri et al. [103], presenting the phytosynthesis of AuNP, their characterization, and application for the degradation of 4-nitrophenol from industrial wastewater, alongside their insecticidal activity.

Regarding the phytosynthesis of AuNP, it can be noticed the variation in terms of NP morphology. The study suggested that triangular-shaped AuNPs with small dimensions present the best catalytic properties.

### 2.3. Palladium Nanoparticles (PdNPs)

Palladium represents an important metal, widely used in environmental protection, for numerous catalytic applications [104,105]. Considering its catalytic potential, palladium became in the last years an important research topic, several works describing its phytosynthesis.

In 2014, Khan et al. [106] presented the phytosynthesis of palladium nanoparticles (PdNP) using Pulicaria glutinosa extract, as well as their catalytic application in the Suzuki coupling reaction, obtaining a complete conversion of bromobenzene to biphenyl in under 5 min. The authors assign the reduction of Pd to nanoparticles to the presence of flavonoids and polyphenols of the used extract. Veisi et al. [107] presented in 2015 the phytosynthesis of monodisperse PdNP using herbal tea extract, for the same catalytic application. The authors suggested that flavonoids and polyols play the main role in the NP production. More than that, by conducting the Suzuki reaction open to air, they were able to prove the PdNP stability, while the catalyst was able to support eight catalytic cycles, without any loss in activity. In the same year, Nasrollahzadeh et al. [108] applied phytosynthesized PdNP for Hiyama and Stille cross-coupling reactions, observing a minimum five cycles of reusability for the catalyst. Duan et al. [109] phytosynthesized PdNP using an aqueous extract of *Eucommia ulmoides* bark, obtaining spherical and quasi-spherical nanoparticles, with an average size of 12.2 nm. The obtained NPs showed very good activity for the excellent catalytic activity for the electro-catalytic oxidation of hydrazine and the catalytic reducing degradation of p-aminoazobenzene, alongside a good stability in time in aqueous solutions. 

In 2016, Nasrollahzadeh and Mohammad Sajadi [110] presented the application of phytosynthesized PdNP as a heterogeneous catalyst for the Suzuki-Miyaura coupling reaction, while Tahir et al. [111] studied the influence of phytosynthesis temperature on the dimensions of PdNP and applied the optimized materials to the photodegradation of methylene blue, obtaining a 90% degradation in 70 min. The authors assign the rapid degradation of the dye to small sizes of the PdNP and to the phytoconstituents attached to their surface.

An interesting approach was adopted by Hazarika et al. [112], who used for the phytosynthesis Garcinia pedunculata Roxb as bio-reductant and starch as bio-stabilizer. They obtained small-size, dispersed NPs, with very good catalytic for the Suzuki-Miyaura cross-coupling reaction, alcohol oxidation and Cr(VI) reduction. In the same year, other groups presented the application of phytosynthesized PdNP for the degradation of different compounds (such as 4-nitrophenol or Congo red) [113,114,115] or for selective oxidation of alcohols [116].

In a recent work, Garole et al. [117] presented the phytosynthesis of PdNP assisted by Lagerstroemia speciosa leaves extract. The obtained nanocatalyst (136.5 nm) was used for the reduction of organic pollutants (methylene blue, methyl orange, and 4-nitrophenol) in the presence of sodium borohydride, obtaining complete reduction within 10 min.

### 2.4. Iron Nanoparticles (FeNP)

Njagi et al. [118] used aqueous sorghum bran extracts for the phytosynthesis of amorphous iron nanoparticles (FeNP), which were successfully used for the H_2_O_2_-catalyzed degradation of bromothymol blue. Machado et al. [119] obtained FeNP using black tea, grape marc, and vine leaves aqueous extracts that were applied for the degradation of ibuprofen both from aqueous solutions and sandy soils, obtaining degradation efficiency up to 66% for aqueous solutions (pH dependent) and over 95% for soils.

Lin et al. [120] studied the effect of reaction atmosphere on the dimensions of FeNP phytosynthesized using green tea extract. The authors noticed that larger particles were obtained in oxygen atmosphere, that, through XPS determinations were proved to be predominantly iron oxide in nature. The FeNP obtained in nitrogen atmosphere had smaller dimensions (approximately 84 nm) and had superior capacity for the removal of methylene blue (98.7%, 85.7% within 5 min). 

Using phytosynthesized FeNP, different degradation of different dyes (such as methylene blue, methyl orange, allura red, brilliant blue, green S, or rhodamine B) was achieved by Garole et al. [121], Radini et al. [122], and Khan and Al-Thabaiti [123].

### 2.5. Copper Nanoparticles (CuNP)

Nasrollahzadeh and Sajadi [124] described the phytosynthesis of copper nanoparticles (CuNP) using Ginkgo biloba L. extract and their application for Huisgen [3 + 2] cycloaddition of azides and alkynes at room temperature. The authors obtained spherical nanoparticles, with a narrow size distribution (15–20 nm), and observed over 93% yields for the studied reaction, at 5 h and a concentration of the CuNP of 10 mol%. Prasad et al. [125] obtained, using broccoli extract, CuNP with spherical morphologies and average size of 4.8 nm. The CuNP were successfully applied for the reduction of 4-nitrophenol in the presence of NaBH_4_ (reaction completed within a few minutes with high values of reaction rate constants) and degradation of methylene blue (68% degradation after 28 h) and methyl red (32% degradation after 28 h). At the same time, the authors performed catalyst re-usability studies, observing no significant change in the conversion percent even at the fifth cycle; however, a prolongation of the time necessary to complete the reaction (at the fifth cycle, the time necessary being of 12 min). Superior results were obtained by Nazar et al. [126] for the catalytic degradation of methylene blue (87.11%, after 3 h) using CuNP phytosynthesized via Punica granatum seeds extract.

Nasrollahzadeh et al. [127] used aqueous extract obtained from *Plantago asiatica* to phytosynthesize CuNP with spherical morphology and dimensions between 7-35 nm, in 5 min. The nanoparticles were applied as catalyst for the cyanation of aldehydes, obtaining yields ≥85% (pure isolated product) at reaction times 30–60 min, superior to the classical methods for the synthesis of aryl nitriles.

### 2.6. Bi-Metallic Nanoparticles

Besides the mono-metallic nanoparticles, another important area in nanomaterials phytosynthesis is represented by the bi-metallic nanoparticles. These types of nanoparticles can come in a wide variety of sizes and morphologies [28]. As can be expected, the majority of the studies regarding catalytic applications of bi-metallic nanoparticles are focused on Ag/Au NPs. Ravi et al. [128] described the phytosynthesis of crystalline Ag/AuNP using *Silybum marianum* seed extract as reducing and stabilizing agent. The spherical polycrystalline NPs had dimensions ≤40 nm and exhibited good catalytic performance for the reduction of 4-nitrophenol. The Ag/AuNP (10–20 nm) phytosynthesized by Karthika et al. [63] using bark extract of *Guazuma ulmifolia* L. also showed good reducing capability towards 4-nitrophenol and Congo red. More than that, the bimetallic nanoparticles were found to have the best catalytic properties for the decolorization of 4-nitrophenol, compared with individual nanoparticles.

Bi-metallic Fe/PdNP were obtained by Luo et al. [129] using grape leaf aqueous extract. The nanoparticles exhibited significant catalytic effect for the removal of Orange II dye (98% in 12 h). The kinetic study performed by the authors revealed that the removal fitted to the pseudo-first-order reduction and pseudo-second-order adsorption model, implying that the removal phenomenon involved both adsorption and catalytic reduction.

Al-Asfar et al. [130] phytosynthesized bi-metallic Ag/FeNP using Palm date fruit extract. The nanoparticles presented as disks and with irregular morphologies, with dimensions 5–40 nm. The authors observed a complete decolorization of the bromothymol blue dye in 60 min for the Ag/FeNP/H_2_O_2_ catalytic system. Similar bi-metallic nanoparticles were also obtained by Taghizadeh et al. [131] using *Cupressus sempervirens* extract and their catalytic activity was tested for methyl orange removal, reaching a 98.5% removal after 4 h.

## 3. Metallic Oxides and Chlorides Nanoparticles

Unlike mono-metallic nanoparticles, metal oxides nanoparticles cover a wider range of metals (such as Fe, Cu, Zn, Zr, Ce, Ni, and others). Their phytosynthesis was recently reviewed by Sharma et al. [132], Singh et al. [133], or Basnet et al. [134], covering the plausible mechanisms involved in their formation, as well as potential application in catalysis.

Phytosynthesis of Fe_3_O_4_ was presented by Basavegowda et al. [135]. The authors used aqueous extract of *Artemisia annua* leaves to obtain ferromagnetic nanoparticles, with average diameters of 6.4 nm. Their application as catalyst in organic synthesis led to the synthesis of benzoxazinone and benzthioxazinone derivatives in high yields (over 80%). Vasantharaj et al. [136] proposed the use of *Ruellia tuberosa* leaves extract for the phytosynthesis of hexagonal nanorods Fe_3_O_4_NP. The obtained NPs were used for the degradation of crystal violet dye under solar irradiation, obtaining a degradation of 80% after 150 min reaction. 

Spherical copper oxide nanoparticles (CuONP) were obtained by Nasrollahzadeh et al. [137] in 2015 using an aqueous *Gundelia tournefortii* leaves extract. The nanoparticles were applied for the degradation of 4-nitrophenol (observing the disappearance of the specific 4-nitrophenolate ions UV-Vis peak in 70 seconds after the addition of CuONP) and for the hydration of cyanamides to N-monosubstituted ureas using acetaldoxime (with yields over 88%). Considering the catalyst reusability, the authors observed a decrease in the reaction yield of phenylcyanamide hydration (from 94% for fresh and 1st cycle catalyst to 90% for the 4th cycle). The same group [138] obtained CuONP using *Thymus vulgaris* aqueous leaves extract and applied them for the N-arylation of indoles and amines. The obtained NPs (having diameters under 30 nm) exhibited good catalytic activity (58–98% yields for the arylation of indoles with aryl halides and 86–96% yields for the arylation of amines with aryl iodides). Regarding the catalyst reusability, the authors observed that the catalyst could be used for five times with almost consistent activity (97–92% yield) for the N-arylation of indoles.

Devi et al. [139] obtained CuO and ZnONP using the aqueous extract of *Centella asiatica*, considering as phytosynthesis responsible molecules the polyphenols and protein (or amino acids) constituents of the extract. When applying the obtained NPs as catalyst for the reduction of methylene blue using NaBH_4_, the authors observed a higher catalytic activity of CuONP (rate constant 4.261, compared with 0.311 for ZnONP), that was attributed to their lesser work function value. The catalytic application of phytosynthesized CuONP was also studied by Sathiyavimal et al. [140], Prakash et al. [141], and Gu et al. [142], for the reduction of dyes, such as crystal violet and methyl red, bromothymol blue, and methylene blue or for the production of 3,4-dihydropyrimidinones by Biginelli reaction [141].

Zinc oxide nanoparticles (ZnONP) were phytosynthesized by Suresh et al. [143] using *Artocarpus gomezianus* fruit extract. The authors obtained porous nanoparticles, with dimensions dependent on the concentration of the extract used for the phytosynthesis. The nanoparticles exhibited good catalytic properties for the degradation of methylene blue, especially in alkaline pH, both under direct sunlight and UV light. The degradation of the same dye was studied by Anbuvannan et al. [144] using ZnONP phytosynthesized by aqueous *Phyllanthus niruri* leaves extract, obtaining an almost complete disappearance of the specific UV-Vis peak at 664 nm in under 30 min Patel et al. [145] obtained ZnO nanorods using *Aloe vera* gel and established its catalytic superiority for the thermal decomposition of potassium perchlorate, compared with nanorods of ZnO obtained using a commercial surfactant (poly(ethylene)glycol). 

The same type of nanoparticles, obtained using different plant extracts were successfully applied by Raja et al. [146], Ishwarya et al. [147], and Ali et al. [148] for the photodegradation of dyes (methylene blue and methyl orange), all the authors observing an elevated degradation efficiency, especial towards methylene blue (over 90% degradation). Phukan et al. [149] obtained ZnO nanotapes, with widths within 9 nm using *Lantana camara* flowers extract and applied the nanostructures for *ipso*-hydroxylation of different aryl/ hetero-arylboronic acid to phenol. Khan et al. [150] obtained spherical ZnONP using *Trianthema portulacastrum* plant extract and applied them for the degradation of a textile dye (Synozol Navy Blue-KBF), obtaining a 91% degradation of the dye in 159 min The author also proposed a mechanistic pathway for the dye degradation, involving the physical adsorption of the dye on the nanoparticles surface, followed by the photocatalytic degradation under the action of OH radicals.

Tin oxide nanoparticles (SnO_2_NP) were obtained by Haritha et al. [151] using *Catunaregam spinosa* bark extract. The nanoparticles were applied for the degradation of the Congo red dye, obtaining a 92% degradation after 45 min of reaction. The authors assign the main role in the phytosynthesis to the hydroxyl group containing phytomolecules. Begum and Ahmaruzzaman [152] used for the phytosynthesis of SnO_2_NP using an aqueous extract of the pods of a tropical leguminous tree (*Parkia speciosa* Hassk). The obtained structures were applied for the degradation of the acid yellow 23 dye, reaching a 98% degradation under UV-C irradiation for 24 min and a reusability of five cycles, without any losses in terms of stability and morphology. 

Phytosynthesized titania nanoparticles (TiO_2_NP) were obtained by Thandapani et al. [153] and Udayabhanu et al. [154] and applied for the catalytic degradation of dyes (methylene blue, methyl orange, crystal violet, and alizarin red), obtaining degradation efficiencies over 77% after 6 h. In both studies, the best effect (over 92% degradation) was observed for methylene blue, while the lowest degradation efficiencies were observed for alizarin red (77.3%) and methyl orange (77.5%). Considering the comparable morphologies and sized of the NPs (Table 1), it can be concluded that the differences in terms of catalytic activity are due to the different phytochemicals from the used extracts.

Other types of metallic oxides nanoparticles (such as Mn_3_O_4_NP, CeO_2_NP, ZrONP, or NiONP) were phytosynthesized by different groups [155,156,157,158] and used for different catalytic applications (catalytic thermal decomposition of ammonium perchlorate; degradation of crystal violet; degradation of methyl orange; photocatalytic degradation of 4-clorphenol). 

Although not as common as the metallic oxides, phytosynthesized metallic chlorides are also encountered in the literature. For example, Huo et al. [159] obtained, using aqueous root extract of *Glycyrrhiza uralensis*, silver chloride nanoparticles (AgClNP). The obtained nanoparticles were successfully applied for the degradation of methylene blue, as a model test pollutant.

As many catalytic processes are related to the metal surface, NPs are usually more reactive than the bulk metal, a consequence of their smaller sizes and larger surface areas. Comparing metallic oxides nanoparticles, iron oxide magnetic nanoparticles could be considered a more desirable alternative for real-life applications, as they are easier to recover from the system, by applying an exterior magnetic field.

## 4. Complex Catalytic Structures Based on Phytosynthesized Nanoparticles

Besides the nanoparticles described in the previous chapters, the literature also presents complex structures, incorporating phytosynthesized nanomaterials, starting from the simple M^1^/M^2^_x_N_y_ catalysts (where M^1^ and M^2^ are metals, N = O, Cl) to composites such as membrane or zeolite-metallic nanoparticles catalysts. In the following paragraphs, we will present a short overview of the recent advances registered in this area.

Nasrollahzadeh et al. [160] used aqueous cocoa seeds extract for the phytosynthesis of Pd/CuO nanoparticles with average size of 40 nm, assigning the main role in the phytosynthesis process to different compounds (epicatechin, catechin, flavonoid and phenolic acids) present in the extract. The nanoparticles were applied for the reduction of 4-nitrophenol with NaBH_4_, obtaining a complete disappearance of the specific absorption peak at 400 nm in 60 seconds, with a reusability of over six cycles. The nanoparticles were also proven to be a highly efficient and stable heterogeneous catalyst for phosphine-free Heck coupling reaction. Using aqueous *Silybum marianum* L. seeds extract, Sajadi et al. [161] obtained magnetic Cu/Fe3O4NP used as catalysts for the reduction of various nitroarenes (with yields of 88-97% and a recyclability of at least five cycles). Nasrollahzadeh and Sajadi [162] applied phytosynthesized Pd/TiO_2_NP as a catalyst for the ligand-free Suzuki–Miyaura coupling reaction (registering yields up to 98% and a four cycles reusability without a significant yield decrease). Reduction of different compounds (4-nitrophenol, methyl orange, Congo red, and methylene blue) was achieved by Atarod et al. [163] and Momeni et al. [164] in 2016, using phytosynthesized Ag/TiO_2_NP and Cu/ZnONP, with very low reaction time. 

Sajadi et al. [165] applied phytosynthesized Ag/Fe_3_O_4_NP for the [2+3] cycloaddition of arylcyanamides and sodium azide, while Hag et al. [166] used Ag/NiONP for the catalytic degradation of rhodamine B. 

Ag/AgCl nanoparticles were obtained by Devi et al. [167,168] using *Momordica charantia* and *Benincasa hispida* extracts. The authors applied the nanoparticles for the reduction of 2-, 4-dinitrophenyl hydrazine for the degradation of malachite green oxalate under sunlight irradiation. 

Other types of phytosynthesized catalytic material are represented by the metal oxide/metal oxide nanostructures. For example, Bharati and Suresh [169] proposed the application of phytosynthesized ZnO/SiO_2_ nanostructures for the reduction of acenaphthylene from refinery waste water, while Maria Magdalane et al. [170] used CeO_2_/CdO multilayered nanoplatelet for the degradation malachite green and catalytic hydrogenation of 4-nitrophenol. 

Using aqueous *Opuntia dilenii haw*, *Azadirachta indica*, *Ocimum sanctum,* and *Saraca indica* extracts, Kombaiah et al. [171] and Garg et al. [172] obtained nano-ferrite (ZnFe_2_O_4_) and bismuth oxychloride (BiOCl), and demonstrated their potential for the oxidation of glycerol into formic acid for the degradation of methyl orange and bisphenol A.

Another widely encountered strategy is represented by the incorporation of phytosynthesized in porous materials (especially zeolites). For example, Zhan et al. [173] reported the obtaining of a AuNP/ titanium silicalite-1 composite for vapor phase propylene epoxidation, Nasrollahzadeh et al. [174] reported the *N*-formylation of amines using CuNP/zeolite (Natrolite) as a “green” catalyst, and Hatamifard et al. [175] used AgNP/zeolite nanocomposite for the ligand-free hydroxylation of phenylboronic acid to phenol and the reduction of 4-nitrophenol (4-NP), methyl orange (MO), Congo red (CR), methylene blue (MB) and rhodamine B, while Das et al. [176] applied AgNP/zeolite (mesoporous silicate-1) catalyst for the reduction of 4-nitrophenol. 

Catalytic composites constructed with reduced graphene oxides (RGO) and phytosynthesized nanoparticles were evaluated by Nasrollahzadeh et al. (PdNP/RGO) [177], Atarod et al. (Pd/RGO/Fe_3_O_4_) [178], Maham et al. (Ag/RGO/Fe_3_O_4_) [179], Nasrollahzadeh et al. (Cu/RGO/Fe_3_O_4_) [180], Khan et al. (Pd/RGO) [181], and Anasdass et al. [182] for the reduction of nitroarenes, reduction of 4-nitrophenol, reduction of 4-nitrophenol, Congo red and Rhodamine B, direct cyanation of aldehydes with K_4_[Fe(CN)_6_], as catalyst for the Suzuki-Miyaura coupling, and as catalyst for Suzuki cross-coupling reactions.

Smuleac et al. [183] incorporated mono- and bi-metallic phytosynthesized nanoparticles (Fe and Fe/Pd) in a polyacrylic acid-coated polyvinylidene fluoride membrane and applied them for the degradation of trichloroethylene, observing the preservation of the membrane reactivity after 4 months of use. Momeni et al. [184] applied Ag/bone nanocomposites for the hydration of cyanamides and Goswami et al. [185] used AgNP supported on cellulose for the degradation of methylene blue, methyl orange, bromophenol blue, Eosin Y and Orange G, while Naeimi et al. [186] phytosynthesized MoO_3_ nanoparticles and used them for the construction of MoO_3_/Copper Schiff base complex with application in the oxidation of alcohols.

Another interesting approach, presented by several authors, is represented by the deposition of the phytosynthesized nanoparticles on different supports (by-products or wastes from different industries). Rostami-Vartooni [187] obtained CuONP deposited on seashell surface and applied the composites for the reduction of 4-nitrophenol and Congo red in the presence of NaBH_4_, while Bordbar and Mortazavimanesh [188] applied PdNP/walnut shell composites for the reduction of 4-nitrophenol, Congo red, methylene blue, and rhodamine B, with reaction times under 1 min Khodadadi et al. [189] deposited PdNP on apricot kernel shells and used them as catalysts for the reduction of 4-nitrophenol, methyl orange, methylene blue, rhodamine B, and Congo red at room temperature (reaction completed in seconds); the same group [190] applied the developed strategy for obtaining AgNP/ peach kernel shells, for the reduction of 4-nitrophenol, methyl orange, and methylene blue (reaction times under 15 min).

**Figure 2:**
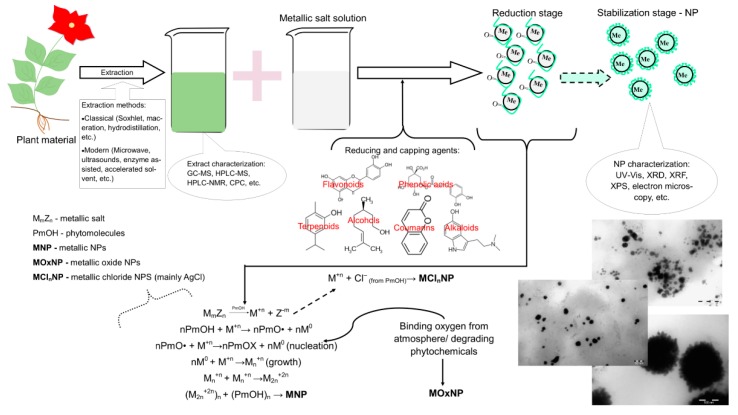
General procedure and phytosynthesis mechanisms for obtaining the “green” nanocatalysts (adapted from [28,32,127] and [167] and for the characterization steps (Transmission electron microscopy-TEM images of silver-AgNP, gold-AuNP and gold/silver nanoparticles-Ag/AuNP from the authors’ unpublished results).

The general procedure for developing the presented phytosynthesized nanocatalysts (as concluded from the reviewed articles) and the synthesis mechanisms are presented in Figure 2.

**Figure 3:**
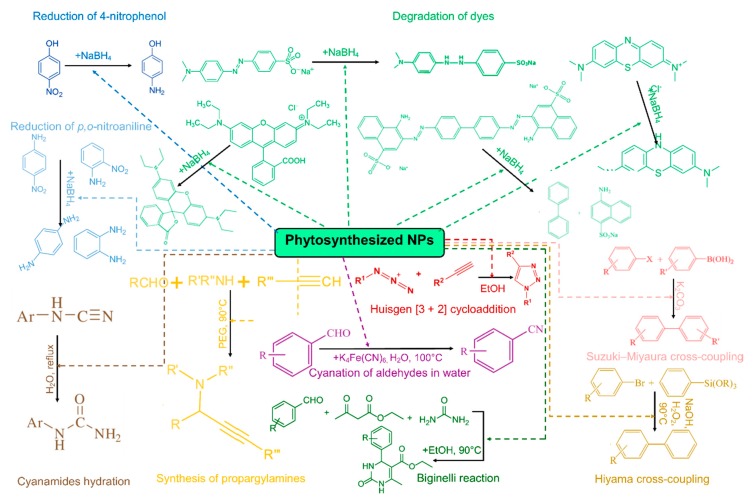
Some of the catalytic applications of the phytosynthesized nanoparticles, emerging from the present review.

Table 1 presents the main applications of phytosynthesized nanoparticles in the field of catalysis, including the extracts used for phytosynthesis, applied characterization techniques, and NPs characteristics, for quick reference. The schematic representation of some of the catalytic applications of nanoparticles identified in the present review is presented in Figure 3.

## 5. Conclusions and Perspectives

From all the presented examples, it can be concluded that the application of phytosynthesized nanoparticles represents a promising area of research in catalysis. When dealing with this type of nanoparticles, the authors should pay special attention to the complete characterization of the obtained materials. UV-Vis spectrometry can provide an important insights on the formation and dimensions of the nanoparticles (by the position of the specific absorbance peaks, as is the case, for example, for AuNP, CuNP, or AgNP, or by the disappearance of adsorption peaks specific for the metal salt, as is the case for Pd, for example). However, the formation of the nanoparticles should be confirmed by other techniques, such as XRD or selected area electron diffraction (SAED) (for the study of their crystalline structure, as well as for the calculation of crystallite size). As for catalytic studies, the size and shape of nanoparticles represents an important parameter, morphology studies should be employed in all studies (most appropriate being TEM, allowing both morphological studies and determination of size distribution of the NP). The main disadvantage of other techniques applied for the determination of particle size (such as Dynamic light scattering (DLS)) is that the technique offers the hydrodynamic diameter, not the “real” diameter of the nanoparticles.

The extracts used for the phytosynthesis also play an important role in the final application. Different types of extraction procedures lead to variations in the composition of the extracts, leading to different sizes and morphologies, thus influencing the catalytic application. Multiple extraction techniques (classical temperature extraction, microwave-assisted, ultrasounds assisted, and accelerated solvent extraction, just to name few examples) could and should be used, in order to optimize the phytosynthesis for the desired application. This observation leads to the major bottleneck in the phytosynthesis of the nanoparticles: although various groups proposes as responsible molecules the phenolic compounds, the flavonoids, or other biomolecules, very few articles applies the fractionation of the extracts and compounds separation, in order to fully elucidate the reaction mechanisms and the biomolecules acting as reducing and capping agents.

Considering the catalytic applications, most of the studies demonstrate the application of NP in synthetic aqueous matrixes, not in real conditions. However, in order to reach this step, the proposed catalytic structures should be incorporated in water treatment installations, easily to separate from the water sources and to prove their re-usability. In spite the depicted drawbacks, we consider the area of NP application in catalysis as a promising research field, which could provide important technological advances and knowledge. It is important to get a thorough understanding of all of the aspects regarding specific interaction of biomolecules with inorganic materials in order to produce NPs with hierarchical structures. The quest of elucidating the specificity of a biomolecule and its adsorption on the surface of a particle still remain a challenge. Additionally, in the future, the plant extract-based catalyst preparation should focus on using vegetal wastes since they contain the same organic agent like raw materials and therefore the circle of sustainability can be complete.

## Figures and Tables

**Table 1:** Examples of the phytosynthesized nanoparticles, applied characterization techniques and their catalytic applications ^1^.

NPs	Plant Extract Used	NPs Characteristics	Applied Characterization Techniques	Catalytic Activity	Ref.
Ag, Au	*Breynia rhamnoides*	Ag–spherical, 64 nm Au–spherical, 27 nm	UV-Vis, TEM, FTIR, particle size distribution	Catalytic conversion of 4-nitrophenol	[33]
Ag	Aqueous *Saraca indica* flowers extract	Spherical, 18–22 nm	UV-Vis, TEM, XRD, FTIR	Reduction of Methylene blue	[34]
Ag	Aqueous *Alstonia macrophylla* leaf extract	Spherical, 70 nm	UV-Vis, FTIR, XRD, SEM, particle size distribution	Reduction of 4-nitrophenol and *p*-nitroaniline	[35]
Ag	Aqueous *Trigonella foenum-graecum* seeds extract	Spherical and quasi-spherical, 22–32 nm	UV-Vis, XRD, TEM, FTIR	Catalytic degradation of methyl orange, methylene blue and eosin Y	[36]
Ag	Aqueous *Euphorbia condylocarpa M. bieb* root extract	Spherical, diameters not determined	UV-Vis, FTIR, TEM	Catalytic synthesis of N-monosubstituted ureas in water	[37]
Ag	*Coleus forskohlii* root extract	Triangular and spherical, 30–40 nm and 35–55 nm, depending on the extract quantity	UV-Vis, XRD, TEM, FTIR	Catalytic reduction of 4-nitrophenol	[38]
Ag	Aqueous *Salicornia brachiate* extract	Spherical, rod-like, prism, triangular, pentagonal and hexagonal, 30–40 nm	UV-Vis, SEM, TEM	Catalytic reduction of 4-nitrophenol	[39]
Ag	Aqueous *Leucas martinicensis* leaves extract	Nearly spherical 20–30 nm	UV-Vis, XRD, TEM, FTIR	Reduction of methylene blue	[40]
Ag	Aqueous *Tribulus terrestris* extract	Spherical, 15–40 nm	UV-Vis, XRD, TEM, FTIR	Reduction of methylene blue	[41]
Ag	Aqueous beetroot extract	Spherical, 15 nm	UV–Vis, XRD, TEM	Reduction of 4-nitrophenol to 4-aminophenol	[42]
Ag, Au	Aqueous *Aerva lanata* leaves extract	Ag–Quasi-spherical, 18.62 nm Au–different morphologies, 17.97 nm	UV-Vis, FTIR, XRD, TEM	Reduction of 4-nitrophenol to 4-aminophenol	[43]
Ag	Aqueous *Ziziphus Jujuba* leaves extract	Different shapes, 20–30 nm, hydrodynamic size 28 nm	UV-Vis, XRD, FT-IR, TEM, DLS, Zeta potential	Reduction of 4-nitrophenol and methylene blue	[44]
Ag	Aqueous *Momordica charantia* leaves extract	Spherical, 16 nm	XRD, SAED, SEM, XPS, UV-Vis, FTIR	Reduction of methylene blue	[45]
Ag	Aqueous *Lantana camara* leaves extract	Almost spherical, 23–30 nm, varying with silver precursor concentration	XRD, SEM, TEM, UV-Vis, FTIR, Zeta potential	Reduction of methylene blue	[46]
Ag	Ferredoxin–NADP+ reductase/ferredoxin obtained from spinach extract	Spherical, 10–15 nm	UV-Vis, TEM, FTIR	Degradation of methylene blue, methyl orange and methyl red	[47]
Ag	Aqueous extracts of *Mentha arvensis* var. *piperascens*, *Buddleja officinalis* Maximowicz, *Epimedium koreanum* Nakai, *Artemisia messer-schmidtiana* Besser, *Magnolia kobus*	Average particle sizes 24.7 to 40 nm	EDS, SEM, EDX, XPS, TEM	Degradation of cellulose by cellulase	[48]
Ag	Aqueous extract from dried *Euphorbia helioscopia* Linn leaves	Spherical, 2–14 nm	TEM, XRD, FT-IR, UV-Vis	Synthesis of propargylamines	[49]
Ag	Aqueous bark extract of *Terminalia cuneata*	Distorted spherical shape, 25–50 nm	FTIR, XRD, DLS, TEM, EDX	Reduction of direct yellow-12 dye	[51]
Ag	Aqueous extract of *Erigeron bonariensis*	Spherical, average size 13 nm	SEM, TEM, XRD, SAED, AFM, FTIR, UV-Vis	Degradation of Acridine Orange	[52]
Ag	Aqueous extract of *Anacardium* *occidentale* testa	Distorted spherical shape, 25 nm	UV-Vis, FTIR, XRD, TEM	Degradation of Congo red and methyl orange	[53]
Ag	Aqueous extract of *Crotolaria retus* leaves	Spherical, 80 nm	UV-Vis, TEM, DLS	Reduction of cresyl blue	[54]
Ag	Aqueous extract of Lychee (*Litchi Chinensis*) fruit peel	Spherical, 4–8 nm	UV-Vis, XRD, EDX, SAED, TEM, FTIR	Photocatalytic degradation of methylene blue	[55]
Ag	Aqueous extract of *Guiera senegalensis*	Spherical, 50 nm	FTIR, TEM, SEM, XRD, EDX	Degradation of 4-nitrophenol and Congo red	[56]
Ag	Aqueous extract of *Cicer arietinum* leaves	Spherical, 88.8 nm	UV-Vis, SEM, TEM, DLS, EDX, FTIR,	Degradation of 4-nitrophenol, methylene blue and Congo red	[57]
Ag, Au	Aqueous *Aglaia elaeagnoidea* flower extract	Ag–spherical, 17 nm Au–spherical, 25 nm	UV–Vis, FTIR, XRD, SEM, EDX, TEM	Degradation of 4-nitrophenol, methylene blue and Congo red	[58]
Ag	Aqueous *Phoenix Dactylifera* extract	Spherical, 25–60 nm	UV-Vis, XRD, FTIR, SEM, TEM, AFM, EDX, Zeta potential	Degradation of 4-nitrophenol	[59]
Ag, Au	Aqueous leaf extract of *Mussaenda glabrata*	Ag–spherical, 51.32 nm Au–triangular and spherical, 10.59 nm	UV-Vis, FTIR, XRD, EDX, SAED, TEM, AFM	Degradation of 4-nitrophenol, rhodamine B and methyl orange	[60]
Ag	Aqueous leaf extract of *Cascabela thevetia* and *Wrightia tomentosa*	Not determined	UV-Vis	Degradation of 4-nitrophenol, methylene blue, methyl orange and methyl red	[61]
Ag	Fractionated *Cassia auriculata* L. flower aqueous extract	Spherical and triangular, 10–35 nm	UV-Vis, FTIR, XRD, TEM	Degradation of 4-nitrophenol, methyl orange	[62]
Ag, Au, Ag/Au	Aqueous bark *Guazuma ulmifolia* L. extract	Ag–spherical, 10–15 nm Au–spherical, 20–25 nm Ag/Au–spherical, 10–20 nm	UV-Vis, FTIR, XRD, AFM, TEM	Degradation of 4-nitrophenol, Congo red	[63]
Ag	90% ethanol extract of *Dillenia indica* bark	Spherical, 15–35 nm	UV-Vis, XRD, TEM, FTIR	Degradation of 4-nitrophenol, methylene blue	[64]
Ag	*Terminalia bellirica* fruit aqueous extract	Spherical, ≤20.6 nm	UV–Vis, FTIR, Zetasizer, SEM, EDX, XRD	Degradation of 4-nitrophenol	[65]
Ag, Au	*Actinidia deliciosa* fruit extract	Ag–spherical, 25–40 nm Au–spherical, 7–20 nm	EDAX, XPS, XRD, FTIR	Degradation of 4-nitrophenol, methylene blue	[66]
Ag	Aqueous extract of *Camellia japonica* leaves	Spherical, 12–25 nm	UV-Vis, XRD, FTIR, TEM, EDX	Photocatalytic degradation of eosin-Y	[67]
Ag	Aqueous extract of *Caulerpa serrulate* algae	Spherical, 10 ± 2 nm	UV–Vis, FT-IR, XRD, TEM	Degradation of Congo red	[68]
Ag	*Artemisia tournefortiana* Rchb ethanol extract	Spherical, average diameter 22.89 ± 14.82 nm	UV-Vis, TEM, SEM, EDX, XRD, FTIR	Degradation of Coomassie Brilliant Blue G-250 under UV light	[69]
Ag	Aqueous bark extract of *Acanthopanax sessiliflorus*	Roughly-spherical, average diameter ~20 nm	UV-Vis, XRD, TEM	Reduction of *p*-nitroaniline to *p*-phenylenediamine	[70]
Ag	Aqueous *Gongronema latifolium* leaf extract	Spherical, 12–30 nm	XRD, SEM, EDS, TEM, UV-Vis, FTIR	Synthesis of *N*-monosubstituted ureas via the hydration of cyanamides in aqueous medium	[71]
Ag	Aqueous extract of *Radix Puerariae*	Spherical, 10–35 nm	TEM, XRD, SEM, DLS, EDX, UV-Vis	Construction of pyrimido[1,2-b]indazole derivatives under solvent-free conditions	[72]
Ag	Aqueous extract of Aqueous palm date fruit pericarp extract	Spherical, 3–30 nm	SEM, TEM, SAED, EDX, DLS	Catalytic degradation of 4-Nitrophenol	[73]
Ag	*Prosopis juliflora* bark extract	Spherical, 10–50 nm	UV-Vis, DLS, SEM, FTIR	Catalytic degradation of 4-Nitrophenol	[74]
Ag, Au	Aqueous leaf extract of *Indigofera tinctoria*	Ag–spherical, 9–26 nm Au–spherical, triangular, hexagonal, 6–29 nm	UV-vis, FTIR, XRD, TEM, EDX, AFM	Reduction of *o/p*-nitroanilines	[75]
Ag	Leaf extract of *Orthosiphon aristatus*	Spherical, 15–45 nm	UV-Vis, FTIR, XRD, TEM, EDX	Reduction of 4-nitrophenol and *o-/p-* nitroaniline	[76]
Ag	Aqueous extract of *Elephantopus scaber*	Spherical, 20–60 nm	UV-Vis, FTIR, XRD, TEM, EDX, AFM	Reduction of 4-nitrophenol, 2-/4-nitroaniline and eosin Y	[77]
Ag, Au	Aqueous extract of *Stemona tuberosa* Lour	Ag–spherical, 25 nm Au–irregular, 20–30 nm	UV-Vis, EDX, XRD, FTIR, SEM, TEM, SAED, Zeta potential	Degradation of 4-nitrophenol, methylene blue, methyl orange and methyl red	[78]
Ag, Au	Aqueousleaves extract of *Myxopyrum serratulum* A. W. Hill	Ag–spherical, 20–50 nm Au–irregular, 6–29 nm	UV-Vis, FTIR, XRD, TEM, EDX	Reduction of 4- nitrophenol, methylene blue and Congo red	[79]
Ag	Aqueous extract of Longan fruit peel	Spherical, 20 nm	UV-Vis, XRD, EDX, TEM, FTIR	Degradation of methylene blue	[80]
Ag	Aqueous extract of *Durio Zibethinus* seeds	Spherical, rod shaped, 20–75 nm	UV-Vis, SEM, TEM, Zeta potential, XRD, EDX	Degradation of methylene blue	[81]
Ag	Aqueous *Convolvulus arvensis* leaves extract	Spherical, 10–30 nm	UV-Vis, SEM, TEM, EDX, XRD, DLS, Zeta potential	Reduction of Reactive Black 5, methyl orange, direct yellow-142	[82]
Ag	Aqueous seed extract of *Gardenia jasminoides* Ellis	Spherical, average size 20 nm	UV-Vis, TEM, XRD, EDX, particle size analyses, FTIR	Reduction of Coomassie Brilliant Blue	[83]
Ag	Aqueous extract of *Allium cepa*	Spherical, 50–100 nm	UV-Vis, SEM, TEM, Particle size, Zeta potential, EDX, XRD, FTIR	Degradation of methylene blue, eosin yellowish, safranin, direct dye, reactive dyes	[84]
Ag	Aqueous *Nervalia zeylanica* leaves extract	Spherical, average particle size 34.2 nm	UV-Vis, FTIR, XRD, AFM, EDX, TEM	Degradation of methyl orange, rhodamine B	[85]
Ag	Aqueous extract of *Bauhinia tomentosa* Linn. leaves	Spherical, 8–25 nm	UV-Vis, XRD, FTIR, SEM, EDX, TEM	Degradation of Rose Bengal	[86]
Ag	Aqueous rhizome extract of *Acorus calamus*	Spherical, hydrodynamic diameter 31.83 nm	UV-Vis, TEM, EDX, FTIR, zeta potential, DLS	Degradation of nitrophenol, 3-nitrophenol, 2, 4, 6-trinitrophenol, picric acid, Coomassie Brilliant Blue, Congo red, eosin Y, rhodamine B, methylene blue, methyl red, methyl orange, cresol red, acridine orange, eriochrome black T, and phenol red	[87]
Ag	Aqueous *Ginkgo biloba* leaves extract	Spherical, 20–40 nm	UV-Vis, XRD, XPS, SEM, TEM, DLS	Reduction of 4-nitrophenol, Congo red, methyl orange, and rhodamine B	[88]
Ag, Au	Aqueous leaf extract of *Bauhinia purpurea*	Ag–quasi-spherical Au–spherical, hexagonal, triangular, nanorods	UV-Vis, FTIR, XRD, TEM, EDX	Degradation of methylene blue and rhodamine B	[89]
Ag	Aqueous *Eriobotrya japonica* (Thunb.) leaves extract	Spherical, 9.26 ± 2.72, 13.09 ± 3.66, and 17.28 ± 5.78 nm, temperature dependent	UV-Vis, SEM, XRD, TEM, FTIR, EDX, SAED	Degradation of Reactive Red 120 and Reactive Black 5	[90]
Au	Aqueous extract of green tea leaves	Mostly spherical, 20 nm	UV-Vis, TEM, XRD	Degradation of methylene blue	[91]
Au	Aqueous extract of *Gnidia glauca* flower	Varying morphology, mostly spherical, 10 nm, with particles up to 150 nm	UV-Vis, TEM, DLS, XRD, FTIR	Degradation of 4-nitrophenol	[92]
Au	70% ethanol *Phoenix dactylifera* L. leaves extract	Spherical, 32–45 nm	UV-Vis, TEM, FTIR, AAS	Degradation of 4-nitrophenol	[93]
Au	Aqueous *Salicornia brachiata* extract	Spherical, 22–35 nm	UV-Vis, SEM, EDX, TEM, XRD	Degradation of 4-nitrophenol and methylene blue	[94]
Au	Aqueous leaf extract of *Polygonum minus*	Mostly icosahedral, 23 nm	UV-Vis, XRD, FTIR, TEM, EDX	Degradation of 4-nitrophenol	[95]
Au	Aqueous *Gnidia glauca* leaves and stem extracts	Mostly spherical, 10–60 nm	UV-Vis, TEM, DLS, XRD, FTIR	Degradation of 4-nitrophenol	[96]
Au	Aqueous *Artemisia capillaris* extracts, different conditions	Spherical, 16.88 ± 5.47~29.93 ± 9.80 nm	UV-Vis, TEM, particle dimension, zeta potential, XRD, FTIR	Degradation of 4-nitrophenol	[97]
Au	Aqueous *Delonix regia* leaves extract	Spherical, 4–24 nm	UV-Vis, TEM, XRD, EDX, SEM, FTIR	Degradation of *o*-nitroaniline	[98]
Au	Aqueous *Lagerstroemia speciosa* leaves extract	Triangular and hexagonal, some spherical, 41–91 nm	TEM, UV-Vis, XRD, FTIR	Reduction of methylene blue, methyl orange, bromophenol blue, bromocresol green, and 4-nitrophenol	[99]
Au	Aqueous *Cotoneaster horizontalis* leaves	Spherical, 18 ± 2 nm	UV-Vis. XRD, TEM, FTIR, SEM, EDX	Degradation of 4-nitrophenol	[100]
Au	Aqueous *Sueda fruciotosa* extract	Spherical, 2–12 nm	UV-Vis, FTIR, XRD, TEM	Degradation of methylene blue	[101]
Au	Ethanol *Artemisia dracunculus* extract	Spherical, hexagonal, and triangular shapes, depending on phytosynthesis conditions, wide variety of dimensions	UV-Vis, SEM, DLS, zeta potential, FTIR	Reduction of 4-nitrophenol	[102]
Pd	Aqueous *Pulicaria glutinosa* extract	Spherical, 20–25 nm	UV-Vis, XRD, TEM, EDX, FTIR	Catalytic conversion of bromobenzene to biphenyl	[106]
Pd	Aqueous *Stachys lavandulifolia* extract	Nearly spherical, 5–7 nm	UV-Vis, TEM, XRD, FTIR, SEM, EDX, ICP	Suzuki reaction, for a wide range of aryl halides	[107]
Pd	Aqueous extract of leaves of *Euphorbiathymifolia* L.	Spherical, 20–30 nm	XRD, TEM, FTIR, UV-vis	Catalyst for Stille and Hiyama cross-coupling reactions	[108]
Pd	Aqueous extract *Eucommia ulmoides* bark	Spherical and quasi-spherical, 10–20 nm	TEM, EDX, XRD,	Electro-catalytic oxidation of hydrazine and catalytic degradation of *p*-aminoazobenzene	[109]
Pd	Aqueous *Euphorbia granulate* leaves extract	25–35 nm	FTIR, TEM	Catalyst for the Suzuki-Miyaura coupling reaction	[110]
Pd	Aqueous *Sapium sebiferum* leaves extract	Spherical, 2–14 nm, depending on the synthesis temperature	UV-Vis, XRD, TEM, SAED, TGA, DLS, FTIR	Photodegradation of methylene blue	[111]
Pd	Aqueous *Garcinia pedunculata* Roxb and starch	Spherical and non-spherical, 2–4 nm	FTIR, XRD, TEM, XPS, SEM, EDX, UV-Vis, TGA, DLS	Catalyst for Suzuki-Miyaura cross-coupling reaction, alcohol oxidation and Cr(VI) reduction	[112]
Pd	Aqueous *Santalum album* extract	Spherical, 10–40 nm	UV-Vis, TEM, XRD, FTIR	Catalytic reduction of 4-nitrophenol	[113]
Pd	Aqueous *Camellia sinensis* leaves extract	Near spherical, 5–8 nm	UV-Vis, XRD, FTIR, SEM, TEM, EDS	Catalytic reduction of 4-nitrophenol, heterogeneous catalyst for Suzuki coupling reactions	[114]
Pd	Aqueous *Pimpinella Tirupatiensis* leaves extract	Spherical, 12.25 nm	UV-Vis, XRD, FTIR, TEM	Catalytic degradation of Congo red	[115]
Pd	Aqueous *Origanum vulgare* L. extract	Spherical, 2–20 nm	UV-Vis, FTIR, XRD, TEM, EDX, TGA	Selective oxidation of alcohols	[116]
Pd	Aqueous *Lagerstroemia speciosa* leaves extract	136.5 nm	UV-Vis, SEM, EDX, XRD, FTIR, TGA	Reduction of methylene blue, methyl orange and 4-nitrophenol	[117]
Fe	Aqueous sorghum bran	Spherical, 40–50 nm	UV-Vis, SEM, TEM, XRD, zeta potential	Degradation of Bromothymol Blue	[118]
Fe	Black tea, grape marc, and vine leaves aqueous extracts	15–45 nm	UV-Vis, TEM	Degradation of ibuprofen	[119]
Fe	Aqueous green tea extract	Spherical, 84.7 ± 11.5 nm and 141.2 ± 26.3 nm, depending on the synthesis atmosphere	SEM, XPS, FTIR	Degradation of methylene blue	[120]
Fe	Aqueous *Lagerstroemia speciosa* leaves extract	Spheroidal, 50–100 nm	UV-Vis, FTIR, EDX, SEM, XRD, TGA	Degradation of methylene blue, methyl orange, allura red, brilliant blue, green S dyes	[121]
Fe	Aqueous extract of *Trigonella foenum-graecum* seed	7–14 nm	UV-Vis, XRD, FTIR, TGA/DTG, TEM, magnetization,	Degradation of methyl orange	[122]
Fe	*HibiscusSabdariffa* flower aqueous extract	Spherical, 18–44 nm, depending on the iron salt concentration	UV-Vis, TEM, FTIR	Degradation of rhodamine B	[123]
Cu	Aqueous *Ginkgo biloba* L. extract	Spherical, 15–20 nm	TEM, EDX, FTIR, UV-Vis	Huisgen [3 + 2] cycloaddition of azides and alkynes	[124]
Cu	Aqueous broccoli extract	Spherical, average particle size 4.8 nm	UV-Vis, FTIR, TEM, DLS, XRD, cyclic voltammetry	Reduction of 4-nitrophenol, degradation of methylene blue and methyl red	[125]
Cu	Aqueous *Punica granatum* seeds extract	Spherical, 40–80 nm, average size 43.9	UV-Vis, XRD, SEM, EDX, FTIR, AFM	Degradation of methylene blue	[126]
Cu	Aqueous *Plantago asiatica* leaf extract	Spherical, 7–35 nm	FTIR, UV–Vis, TEM, XRD	Cyanation of aldehydes usingK_4_Fe(CN)_6_	[127]
Ag/Au	Aqueous *Silybum marianum* extract	Spherical, ≤40 nm	UV-Vis, DLS, XRD, TEM, SAED, EDX, XPS, FTIR	Reduction of 4-nitrophenol	[128]
Fe/Pd	Grape leaf aqueous extract	Quasi-spherical, 10–100 nm	SEM, FTIR	Removal of Orange II	[129]
Ag/Fe	Aqueous Palm dates fruit extract	Disks, irregular, 5–40 nm	UV-Vis, TEM, EDX	Degradation of bromothymol blue	[130]
Ag/Fe	*Cupressus sempervirens* extract	Core-shell, 22–72 nm, mean particle size 36.7 nm	UV-Vis, TEM, XRD, FTIR	Degradation of methyl orange	[131]
Fe_3_O_4_	Aqueous *Artemisia annua* leaves extract	Spherical, 3–10 nm	UV-Vis, XRD, TEM, EDX, FTIR, TGA vibrating sample magnetometry	Catalytic synthesis of benzoxazinone and benzthioxazinone derivatives	[135]
Fe_3_O_4_	Aqueous *Ruellia tuberosa* leaves extract	Hexagonal nanorods, 20–80 nm	UV-Vis, FTIR, SEM, EDX, TEM, DSC, DLS	Degradation of crystal violet	[136]
CuO	Aqueous *Gundelia tournefortii* leaves extract	Spherical, not determined	UV-Vis, SEM, TEM, EDX, XRD, FTIR	Reduction of 4-nitrophenol and synthesis of N-monosubstituted ureas	[137]
CuO	Aqueous *Thymus vulgaris* leaves extract	Quasi-spherical, ≤30 nm	UV-Vis, TEM, EDX, XRD, FTIR, TGA, DTG	N-arylation of indoles and amines	[138]
CuO, ZnO	Aqueous *Centella asiatica* extract	Spherical, average diameter 7 nm	UV-Vis, XRD, SEM, TEM, DLS, FTIR	Reduction of methylene blue	[139]
CuO	Aqueous *Sida acuta* leaves extract	Nanorods, 50 nm	UV-Vis, FTIR, SEM, EDX, TEM	Reduction of crystal violet and methyl red	[140]
CuO	Aqueous *Cordia sebestena* flowers extract	Spherical, 20–35 nm	UV-Vis, FTIR, SEM, EDX, TEM, XRD, DLS, SAED, zeta potential	Production of 3,4-dihydropyrimidinones by Biginelli reaction, degradation of bromothymol blue	[141]
CuO	Aqueous *Cystoseira trinodis* algal extract	Spherical, 7–9 nm	UV-Vis, FTIR, SEM, EDX, TEM, AFM, Raman, FTIR	Degradation of methylene blue	[142]
ZnO	Aqueous *Artocarpus gomezianus* fruits extract	Spherical, porous, 5–47 nm	XRD, SEM, TEM, UV – Vis	Degradation of methylene blue	[143]
ZnO	Aqueous *Phyllanthus niruri* leaves extract	Quasi-spherical, rectangle, triangle, radial hexagonal, rod shaped, 25.61 nm	UV-Vis, photoluminescence, XRD, SEM, TEM, FTIR,	Degradation of methylene blue	[144]
ZnO	*Aloe vera* gel	Nanorods, 15–20 nm	XRD, TEM, SAED, SEM, FTIR, DTG	Thermal decomposition of potassium perchlorate	[145]
ZnO	Aqueous *Tabernaemontana divaricata* leaves extract.	Spherical, 20–50 nm	XRD, UV-Vis, TEM, FTIR	Degradation of methylene blue	[146]
ZnO	*Ulva lactuca* seaweed extract	Sponge-like asymmetrical shaped, 10–50 nm	XRD, UV-Vis, FTIR, SAED, TEM	Degradation of methylene blue	[147]
ZnO	Aqueous *Conyza canadensis* leaves extract	Spherical, ≤10 nm	UV-Vis, FTIR, XRD, TEM, SEM, EDX	Degradation of methylene blue and methyl orange	[148]
ZnO	Aqueous *Lantana camara* flowers extract	Nanotapes, widths within 9 nm	UV-Vis, XRD, FTIR, TGA, TEM, specific surface area, photoluminescence	*Ipso*-hydroxylation of different aryl/hetero-arylboronic acid to phenol	[149]
ZnO	Aqueous *Trianthema portulacastrum* extract	Spherical, 25–90 nm	UV-Vis, XRD, FTIR, SEM, EDX, TEM, XPS	Degradation of Synozol Navy Blue-KBF	[150]
SnO_2_	Aqueous *Catunaregam spinosa* bark extract	Spherical, average size 47 ± 2 nm	UV-Vis, XRD, FTIR, TEM, EDX	Degradation of Congo red	[151]
SnO_2_	Aqueous *Parkia speciosa* Hassk pods extract	Spherical, average size 1.9 nm	UV-Vis, XRD, FTIR, TEM, EDX, SAED	Degradation of acid yellow 23	[152]
TiO_2_	Aqueous *Parthenium hysterophorus* leaves extracts	Spherical, 20–50 nm	UV-Vis, FTIR, SEM, EDX, XRD	Degradation of methylene blue, methyl orange, crystal violet, alizarin red	[153]
TiO_2_	Aqueous *Euphorbia hirta* leaves extract	Spherical, 20–50 nm	UV-Vis. FTIR, XRD, SEM, EDX	Degradation of methylene blue, methyl orange, crystal violet, alizarin red	[154]
Mn_3_O_4_NP	Aqueous *Azadirachta indica* leaves extract	Spherical, 20–30 nm	XRD, FTIR, XPS, SEM, TEM, specific surface area	Catalytic thermal decomposition of ammonium perchlorate	[155]
CeO_2_NP	Methanolic *Moringa oleifera* peel extract	Spherical, average size 40 nm	UV-Vis, FTIR, XRD, TEM, SAED	Degradation of crystal violet	[156]
ZrONP	Aqueous *Lagerstroemia speciosa* Leaves extract	Tetragonal, few oval, average particle size 56.8 nm	UV-Vis, FTIR, XRD, SEM, TEM	Degradation of methyl orange	[157]
NiONP	Hydroalcoholic *Aegle marmelos* leaves extract	Spherical, 8–10 nm	UV-Vis, XRD, SEM, TEM	Degradation of 4-clorphenol	[158]
AgClNP	Aqueous root extract of *Glycyrrhiza uralensis*	Spherical, 5–15 nm	UV-Vis, TEM, SAED, EDX, XRD, DLS, FTIR	Degradation of methylene blue	[159]
Pd/CuO	Aqueous *Theobroma cacao* L. seeds extract	40 nm	FTIR, EDX, XRD, TEM, UV-Vis	Reduction of 4-nitrophenol, catalyst for Heck coupling reaction under aerobic conditions	[160]
Cu/Fe_3_O_4_	Aqueous *Silybum marianum* L. seeds extract	Spherical, 8.5–60 nm	XRD, TEM, EDX, UV-vis	Catalytic reduction of nitroarenes	[161]
Pd/TiO_2_	Aqueous *Myrtus communis* L. extract	Spherical, 17–25	SEM, TEM, FTIR, UV-Vis, EDX	Catalyst for ligand-free Suzuki-Miyaura coupling reaction	[162]
Ag/TiO_2_	Aqueous *Euphorbia heterophylla* leaves extract	Core-shell, under 24 nm	FTIR, UV-Vis, XRD, SEM	Reduction of 4-nitrophenol, methyl orange, Congo red, methylene blue	[163]
Cu/ZnO	Aqueous *Euphorbia prolifera* leaves extract	Core-shell, spherical CuNP 5–17 nm shell	FTIR, UV-Vis, XRD, SEM, EDX	Reduction of Congo red, methylene blue	[164]
Ag/Fe_3_O_4_	Aqueous *Euphorbia peplus* L. leaves extract	Spherical, 5–10 nm	XRD, SEM, TEM, EDX, FTIR, UV-Vis	Catalyst for the [2+3] cycloaddition of arylcyanamides and sodium azide	[165]
Ag/NiONP	Aqueous *Azadirachta indica* L. leaves extract	Irregular morphologies	XRD, SEM, TGA	Degradation of Rhodamine B	[166]
Ag/AgCl	Aqueous *Momordica charantia* leaves extract	Spherical, average size 15 nm	UV-Vis, FTIR, SEM, EDX, XRD, TEM, SAED, TEM	Reduction of 2, 4-dinitrophenyl hydrazine	[167]
Ag/AgCl	Aqueous *Benincasa hispida* peel extract	Spherical, 25–30 nm	UV-Vis, XRD, FTIR, EDX, SEM, XPS	Degradation of malachite green oxalate	[168]
ZnO/SiO_2_	Aqueous *Butea monosperma* flowers extract	Spherical, 3–45 nm	Specific surface area, SEM, EDX, XRD, FTIR	Reduction of acenaphthylene	[169]
CeO_2_/CdO	Concentrated *Citrus limonum* fruit extract	Nanoplatelets, diameter 10 nm, length 50–100 nm.	XRD, FTIR, SEM, TEM, SAED, UV-Vis, TGA-DTG	Degradation of malachite green and catalytic hydrogenation of 4-nitrophenol	[170]
ZnFe_2_O_4_	Aqueous *Opuntia dilenii haw* extract	Spherical, 23–50 nm for microwave assisted synthesis Spherical, 380–680 nm for classical temperature synthesis	FTIR, UV-Vis, EDX, XRD, Magnetization measurements, DLS	Oxidation of glycerol into formic acid	[171]
BiOCl	Aqueous extracts of *Azadirachta indica*, *Ocimum sanctum* and *Saraca indica* leaves	Nanoflower structures, 50–400 nm, depending on the extract	XRD, XPS, SEM, EDX, TEM, UV-Vis, specific surface area	Degradation of methyl orange and bisphenol A	[172]
AuNP/ titanium silicalite-1	*Cacumen Platycladi* extract	1.7 ± 0.3–4.6 ± 0.5 nm	TEM, specific surface area, TGA, UV-Vis	Propylene epoxidation with H2/O2 mixture	[173]
CuNP/zeolite	Aqueous *Anthemis xylopoda* flowers extract	28.5 ± 3 nm (NPs)	XRD, SEM, XRF, UV-Vis, FTIR, TEM, EDX	*N*-formylation of amines	[174]
AgNP/zeolite	Aqueous *Euphorbia prolifera* leaves extract	Semi-spherical, 15 nm (NPs)	FTIR, XRD, SEM, EDX, TEM, UV-Vis	Catalyst for ligand-free hydroxylation of phenylboronic acid to phenol, reduction of 4-nitrophenol, methyl orange, Congo red, methylene blue, rhodamine B	[175]
AgNP/zeolite	Aqueous extract of *Carambola* fruit	Spherical, 10–15 nm	SEM, TEM, EDX, XRD, FTIR, specific surface area, UV-Vis	Reduction of 4-nitrophenol,	[176]
PdNP/RGO	Barberry fruit extract	Spherical, average size 18 nm	UV-vis, XRD, FTIR, SEM, TEM, EDX	Reduction of nitroarenes	[177]
Pd/RGO/Fe_3_O_4_	Aqueous *Withania coagulans* leaves extract	Spherical, ≤15 nm	FTIR, XRD, UV-Vis, SEM, TEM, EDX, magnetic properties,	Reduction of 4-nitrophenol	[178]
Ag/RGO/Fe_3_O_4_	Aqueous *Lotus garcinii* leaves extract	Spherical, 7–20 nm (AgNP)	FTIR, SEM, EDX, XRD, TEM, UV-Vis	Reduction of 4-nitrophenol, Congo red, Rhodamine B	[179]
Cu/RGO/Fe_3_O_4_	Aqueous *Euphorbia bungei* Boiss leaves extract	50–80 nm	UV-Vis, FTIR, XRD, SEM, EDX	Direct cyanation of aldehydes with K_4_[Fe(CN)_6_]	[180]
Pd/RGO	Aqueous *Pulicaria glutinosa* extract	Triangular, 15–18 nm, spherical, isotropic at higher Pd concentration, 7–8 nm	UV-Vis, TEM, EDX, XRD, FTIR, Raman	Catalyst for the Suzuki-Miyaura Coupling	[181]
Pd/RGO	Aqueous *Ficus carica* fruit extract	Spherical, 0.16 nm	UV-Vis, XRD, FTIR, TEM, Raman	Catalyst for Suzuki cross-coupling reactions	[182]
Fe and Fe/Pd/PAA/PVDF membrane	Aqueous green tea extract	Spherical, 20–30 nm, aggregation 80–100 nm	SEM, EDX, XRD	Degradation of trichloroethylene	[183]
Ag/bone	Aqueous *Myrica gale* L. extract	Spherical, 5–10 nm	XRD, SEM, TEM, EDX, FTIR, UV-Vis	Hydration of cyanamides	[184]
AgNP/cellulose	Aqueous seed extract of *Hibiscus sabdariffa*	Spherical, 4 nm	UV-Vis, XRD, FTIR, TEM, SEM	Degradation of methylene blue, methyl orange, bromophenol blue, Eosin Y, Orange G.	[185]
MoO_3_/Copper complex	Aqueous *Sesbania sesban* extract	Cylindrical, 80 × 30 nm	FTIR, EDX, SEM, TEM, AFM	Oxidation of alcohols	[186]
CuONP/ seashell	Aqueous *Rumex crispus* seeds extract	Spherical, 8–60 nm	FTIR, XRD, SEM, EDX, TEM, UV-Vis	Reduction of 4-nitrophenol and Congo red	[187]
PdNP/walnut shell	Aqueous *Equisetum arvense* L. leaves extract	Spherical, 5–12 nm	FTIR, UV-Vis, photoluminescence, XRD, SEM, EDX, TEM	Reduction of 4-nitrophenol, Congo red, methylene blue, and rhodamine B.	[188]
PdNP/apricot kernel shell	70% ethanol *Salvia hydrangea* extract	Spherical, ≤10 nm	FTIR, SEM, UV-Vis, EDX, TEM, XRD	Reduction of 4-nitrophenol, methyl orange, methylene blue, rhodamine B, and Congo red	[189]
AgNP/ peach kernel shells	Aqueous *Achillea millefolium* L. extract	Spherical, ≤20 nm	FTIR, UV-Vis, XRD, SEM, EDS, TGA-DTG, TEM	Reduction of 4-nitrophenol, methyl orange, and methylene blue	[190]

^1^ Where AAS—Atomic absorption spectroscopy; AFM—Atomic force microscopy; DLS—Dynamic light scattering; DSC—Differential scanning calorimetry; DTG—Differential thermogravimetry; EDX—Energy-dispersive X-ray spectroscopy; FTIR-Fourier-transform infrared spectroscopy; ICP-MS—Inductively coupled plasma mass spectrometry; Raman—Raman spectroscopy; SAED—Selected area electron diffraction; SEM—Scanning electron microscope; TEM—transmission electron microscopy; TGA—Thermogravimetric analysis; UV-Vis—Ultraviolet–visible spectroscopy; XPS—X-ray photoelectron spectroscopy; XRD—X-ray diffraction.

## References

[1] Koltsakis G.C., Kandylas I.P., Stamatelos A.M. (2007). Three-Way Catalytic Converter Modeling and Applications. Chem. Eng. Comm..

[2] Taseidifar M., Khataee A., Vahid B., Khorram S., Joo S.W. (2015). Production of nanocatalyst from natural magnetite by glow discharge plasma for enhanced catalytic ozonation of an oxazine dye in aqueous solution. J. Mol. Catal. A Chem..

[3] Vahid B., Mousanejad T., Khataee A. (2014). Sonocatalytic ozonation, with nano-TiO_2_ as catalyst, for degradation of 4-chloronitrobenzene in aqueous solution. Res. Chem. Intermed..

[4] Rapsomanikis A., Apostolopoulou A., Stathatos E., Lianos P. (2014). Cerium-modified TiO_2_ nanocrystalline films for visible light photocatalytic activity. J. Photochem. Photobiol. A Chem..

[5] Baerns M. (2014). Aspects of Heterogeneous Catalysis and of Its Industrial and Environmental Practice. Reference Module in Chemistry, Molecular Sciences and Chemical Engineering.

[6] Khan I., Saeed K., Khan I. (2017). Nanoparticles: Properties, applications and toxicities. Arab. J. Chem..

[7] Jeevanandam J., Barhoum A., Chan Y.S., Dufresne A., Danquah M.K. (2018). Review on nanoparticles and nanostructured materials: History, sources, toxicity and regulations. Beilstein. J. Nanotechnol..

[8] Holišová V., Urban M., Kolenčík M., Němcová Y., Schröfel A., Peikertová P., Slabotinský J., Kratošová G. (2019). Biosilica-nanogold composite: Easy-to-prepare catalyst for soman degradation. Arab. J. Chem..

[9] Sharma N., Ojha H., Bharadwaj A., Pathak D.P., Sharma R.K. (2015). Preparation and catalytic applications of nanomaterials: A review. RSC Adv..

[10] Chu V.H., Nghiem T.H.L., Le T.H., Vu D.L., Tran H.N., Vu T.K.L. (2012). Synthesis and optical properties of water soluble CdSe/CdS quantum dots for biological applications. Adv. Nat. Sci. Nanosci. Nanotechnol..

[11] Reda S.M. (2008). Synthesis and optical properties of CdS quantum dots embedded in silica matrix thin films and their applications as luminescent solar concentrators. Acta Mater..

[12] Zhang J., Langille M.R., Mirkin C.A. (2011). Synthesis of silver nanorods by low energy excitation of spherical plasmonic seeds. Nano Lett..

[13] Pan D., Wang Q., An L. (2009). Controlled synthesis of monodisperse nanocrystals by a two-phase approach without the separation of nucleation and growth processes. J. Mater. Chem..

[14] Zhang H. (2018). Introduction: 2D Materials Chemistry. Chem. Rev..

[15] Wei F., Zeng H., Cui P., Peng S., Cheng T. (2008). Various TiO_2_ microcrystals: Controlled synthesis and enhanced photocatalytic activities. Chem. Eng. J..

[16] Luo J., Duan G., Wang W., Luo Y., Liu X. (2017). Size-controlled synthesis of palygorskite/Ag_3_PO_4_ nanocomposites with enhanced visible-light photocatalytic performance. Appl. Clay Sci..

[17] Wang J., Li J., Gao M., Zhang X. (2018). Recent advances in covalent organic frameworks for separation and analysis of complex samples. TrAC Trends Anal. Chem..

[18] Nasrabadi H.T., Abbasi E., Davaran S., Kouhi M., Akbarzadeh A. (2016). Bimetallic nanoparticles: Preparation, properties, and biomedical applications. Artif. Cells Nanomed. Biotechnol..

[19] Khan Z., Al-Thabaiti S.A., Obaid A.Y., Khan Z.A., Al-Youbi A.A. (2012). Shape-directing role of cetyltrimethylammonium bromide in the preparation of silver nanoparticles. J. Colloid Interface Sci..

[20] Fierascu R.C., Fierascu I., Lungulescu E.M., Nicula N., Somoghi R., Diţu L.M., Ungureanu C., Sutan A.N., Drăghiceanu O.A., Paunescu A. (2019). Phytosynthesis and radiation-assisted methods for obtaining metal nanoparticles. J. Mat. Sci..

[21] de Souza T.A.J., Souza L.R.R., Franchi L.P. (2019). Silver nanoparticles: An integrated view of green synthesis methods, transformation in the environment, and toxicity. Ecotoxicol. Environ. Safe.

[22] Brusotti G., Cesari I., Dentamaro A., Caccialanza G., Massolini G. (2014). Isolation and characterization of bioactive compounds from plant resources: The role of analysis in the ethnopharmacological approach. J. Pharm. Biomed. Anal..

[23] Halket J.M., Waterman D., Przyborowska A.M., Patel R.K., Fraser P.D., Bramley P.M. (2005). Chemical derivatization and mass spectral libraries in metabolic profiling by GC/MS and LC/MS/MS. J. Exp. Bot..

[24] Sieniawska E., Baja T., Dudka J., Gieroba R., Swiatek L., Rajtar B., Glowniak K., Polz-Dacewicz M. (2013). Cytotoxicity, antioxidant activity and an effect on CYP3A4 and CYP2D6 of Mutellina purpurea L. extracts. Food Chem. Toxicol..

[25] Takla S.S., Shawky E., Hammoda H.M., Darwish F.A. (2018). Green techniques in comparison to conventional ones in the extraction of Amaryllidaceae alkaloids: Best solvents selection and parameters optimization. J. Chromatogr. A.

[26] Akhtar M.S., Panwar J., Yun Y.S. (2013). Biogenic Synthesis of Metallic Nanoparticles by Plant Extracts. ACS Sustain. Chem. Eng..

[27] Siddiqi K.S., Husen A. (2017). Recent advances in plant-mediated engineered gold nanoparticles and their application in biological system. J. Trace Elem. Med. Biol..

[28] Fierascu I., Georgiev M.I., Ortan A., Fierascu R.C., Avramescu S.M., Ionescu D., Sutan A., Brinzan A., Ditu L.M. (2017). Phyto-mediated metallic nanoarchitectures via *Melissa officinalis* L.: Synthesis, characterization and biological properties. Sci. Rep..

[29] Fahmy H.M., Saad O.A., Rashed H.A., Hessen O.E.A., Elgamal K.H.I., Aboelfetouh M.M. (2017). Alternative Green Chemistry Methods of Silver Nanoparticles Synthesis: Review and Comparison. J. Bionanosci..

[30] Hoseinpour V., Ghaemi N. (2018). Green synthesis of manganese nanoparticles: Applications and future perspective–A review. J. Photochem. Photobiol. B.

[31] Khan M., Shaik M.R., Adil S.F., Khan S.T., Al-Warthan A., Siddiqui M.R.H., Tahir M.N., Tremel W. (2018). Plant extracts as green reductants for the synthesis of silver nanoparticles: Lessons from chemical synthesis. Dalton Trans..

[32] Sutan N.A., Manolescu D.S., Fierascu I., Neblea A.M., Sutan C., Ducu C., Soare L.C., Negrea D., Avramescu S.M., Fierascu R.C. (2018). Phytosynthesis of gold and silver nanoparticles enhance in vitro antioxidant and mitostimulatory activity of *Aconitum toxicum* Reichenb. rhizomes alcoholic extracts. Mater. Sci. Eng. C.

[33] Gangula A., Podila R., Ramakrishna M., Karanam L., Janardhana C., Rao A.M. (2011). Catalytic Reduction of 4-Nitrophenol using Biogenic Gold and Silver Nanoparticles Derived from *Breynia rhamnoides*. Langmuir.

[34] Vidhu V.K., Philip D. (2014). Spectroscopic, microscopic and catalytic properties of silver nanoparticles synthesized using *Saraca indica* flower. Spectrochim. Acta A Mol. Biomol. Spectrosc..

[35] Borase H.P., Patil C.D., Salunkhe R.B., Suryawanshi R.K., Salunke B.K., Patil S.V. (2014). Catalytic and synergistic antibacterial potential of green synthesized silver nanoparticles: Their ecotoxicological evaluation on *Poecillia reticulata*. Biotechnol. Appl. Biochem..

[36] Vidhu V.K., Philip D. (2014). Catalytic degradation of organic dyes using biosynthesized silver nanoparticles. Micron.

[37] Nasrollahzadeh M., Babaei F., Sajadi S.M., Ehsani A. (2014). Green synthesis, optical properties and catalytic activity of silver nanoparticles in the synthesis of N-monosubstituted ureas in water. Spectrochim. Acta A Mol. Biomol. Spectrosc..

[38] Naraginti S., Sivakumar A. (2014). Eco-friendly synthesis of silver and gold nanoparticles with enhanced bactericidal activity and study of silver catalyzed reduction of 4-nitrophenol. Spectrochim. Acta A Mol. Biomol. Spectrosc..

[39] Seralathan J., Stevenson P., Subramaniam S., Raghavan R., Pemaiah B., Sivasubramanian A., Veerappan A. (2014). Spectroscopy investigation on chemo-catalytic, free radical scavenging and bactericidal properties of biogenic silver nanoparticles synthesized using *Salicornia brachiata* aqueous extract. Spectrochim. Acta A Mol. Biomol. Spectrosc..

[40] Ashokkumar S., Ravi S., Kathiravan V., Velmurugan S. (2014). Rapid biological synthesis of silver nanoparticles using *Leucas martinicensis* leaf extract for catalytic and antibacterial activity. Environ. Sci. Pollut. Res. Int..

[41] Ashokkumar S., Ravi S., Kathiravan V., Velmurugan S. (2014). Synthesis, characterization and catalytic activity of silver nanoparticles using *Tribulus terrestris* leaf extract. Spectrochim. Acta A Mol. Biomol. Spectrosc..

[42] Bindhu M.R., Umadevi M. (2015). Antibacterial and catalytic activities of green synthesized silver nanoparticles. Spectrochim. Acta A Mol. Biomol. Spectrosc..

[43] Joseph S., Mathew B. (2015). Microwave assisted facile green synthesis of silver and gold nanocatalysts using the leaf extract of *Aerva lanata*. Spectrochim. Acta A Mol. Biomol. Spectrosc..

[44] Gavade N.L., Kadam A.N., Suwarnkar M.B., Ghodake V.P., Garadkar K.M. (2015). Biogenic Synthesis of Multi-applicative Silver Nanoparticles by using Ziziphus Jujuba Leaf Extract. Spectrochim. Acta A Mol. Biomol. Spectrosc..

[45] Ajitha B., Reddy Y.A.K., Reddy P.S. (2015). Biosynthesis of silver nanoparticles using *Momordica charantia* leaf broth: Evaluation of their innate antimicrobial and catalytic activities. J. Photochem. Photobiol. B.

[46] Ajitha B., Reddy Y.A.K., Shameer S., Rajesh K.M., Suneetha Y., Reddy P.S. (2015). *Lantana camara leaf* extract mediated silver nanoparticles: Antibacterial, green catalyst. J. Photochem. Photobiol. B.

[47] Ahmed K.B.A., Senthilnathan R., Megarajan S., Anbazhagan V. (2015). Sunlight mediated synthesis of silver nanoparticles using redox phytoprotein and their application in catalysis and colorimetric mercury sensing. J. Photochem. Photobiol. B.

[48] Salunke B.K., Sawant S.S., Kang T.K., Seo D.Y., Cha Y., Moon S.A., Alkotaini B., Sathiyamoorthi E., Kim B.S. (2015). Potential of Biosynthesized Silver Nanoparticles as Nanocatalyst for Enhanced Degradation of Cellulose by Cellulase. J. Nanomat..

[49] Nasrollahzadeh M., Sajadi S.M., Babaei F., Mahamd M. (2015). Euphorbia helioscopia Linn as a green source for synthesis of silver nanoparticles and their optical and catalytic properties. J. Colloid Interface Sci..

[50] Park J., Cha S.H., Cho S., Park Y. (2016). Green synthesis of gold and silver nanoparticles using gallic acid: Catalytic activity and conversion yield toward the 4-nitrophenol reduction reaction. J. Nanopart. Res..

[51] Edison T.N.J.I., Lee Y.R., Sethuraman M.G. (2016). Green synthesis of silver nanoparticles using *Terminalia cuneata* and its catalytic action in reduction of direct yellow-12 dye. Spectrochim. Acta A Mol. Biomol. Spectrosc..

[52] Kumar V., Singh D.K., Mohan S., Hasan S.H. (2016). Photo-induced biosynthesis of silver nanoparticles using aqueous extract of *Erigeron bonariensis* and its catalytic activity against Acridine Orange. J. Photochem. Photobiol. B.

[53] Edison T.N.J.I., Atchudan R., Sethuraman M.G., Lee Y.R. (2016). Reductive-degradation of carcinogenic azo dyes using *Anacardium occidentale* testa derived silver nanoparticles. J. Photochem. Photobiol. B.

[54] Ahmed S., Annu K.M., Ikram S. (2016). Synthesis of Silver Nanoparticles Using Leaf Extract of *Crotolaria retusa* as Antimicrobial Green Catalyst. J. Bionanosci..

[55] Khan A.U., Yuan Q., Wei Y., Khan Z.H., Tahir K., Khan S.U., Ahmad A., Khan S., Nazir S., Khan F.U. (2016). Ultra-efficient photocatalytic deprivation of methylene blue and biological activities of biogenic silver nanoparticles. J. Photochem. Photobiol. B..

[56] Bello B.A., Khan S.A., Khan J.A., Syed F.Q., Anwar Y., Khan S.B. (2017). Antiproliferation and antibacterial effect of biosynthesized AgNps from leaves extract of *Guiera senegalensis* and its catalytic reduction on some persistent organic pollutants. J. Photochem. Photobiol. B..

[57] Arya G., Sharma N., Ahmed J., Gupta N., Kumar A., Chandra R., Nimesh S. (2017). Degradation of anthropogenic pollutant and organic dyes by biosynthesized silver nano-catalyst from *Cicer arietinum* leaves. J. Photochem. Photobiol. B.

[58] Manjari G., Saran S., Arun T., Devipriya S.P., Rao A.V.B. (2017). Facile Aglaia elaeagnoidea Mediated Synthesis of Silver and Gold Nanoparticles: Antioxidant and Catalysis Properties. J. Clust. Sci..

[59] Farhadi S., Ajerloo B., Mohammadi A. (2017). Green Biosynthesis of Spherical Silver Nanoparticles by Using Date Palm (*Phoenix Dactylifera*) Fruit Extract and Study of Their Antibacterial and Catalytic Activities. Acta Chim. Slov..

[60] Francis S., Joseph S., Koshy E.P., Mathew B. (2017). Green synthesis and characterization of gold and silver nanoparticles using *Mussaenda glabrata* leaf extract and their environmental applications to dye degradation. Environ. Sci. Pollut. Res..

[61] Karthik R., Govindasamy M., Chen S.M., Cheng Y.H., Muthukrishnan P., Padmavathy S., Elangovan A. (2017). Biosynthesis of silver nanoparticles by using *Camellia japonica* leaf extract for the electrocatalytic reduction of nitrobenzene and photocatalytic degradation of Eosin-Y. J. Photochem. Photobiol. B..

[62] Muthu K., Priya S. (2017). Green synthesis, characterization and catalytic activity of silver nanoparticles using *Cassia auriculata* flower extract separated fraction. Spectrochim. Acta A Mol. Biomol. Spectrosc..

[63] Karthika V., Arumugam A., Gopinath K., Kaleeswarran P., Govindarajan M., Alharbi N.S., Kadaikunnan S., Khaled J.M., Benelli G. (2017). *Guazuma ulmifolia* bark-synthesized Ag, Au and Ag/Au alloy nanoparticles: Photocatalytic potential, DNA/protein interactions, anticancer activity and toxicity against 14 species of microbial pathogens. J. Photochem. Photobiol. B.

[64] Mohanty A.S., Jena B.S. (2017). Innate catalytic and free radical scavenging activities of silver nanoparticles synthesized using *Dillenia indica* bark extract. J. Colloid Interface Sci..

[65] Patil S., Chaudhari G., Paradeshi J., Mahajan R., Chaudhari B.L. (2017). Instant green synthesis of silver-based herbo-metallic colloidal nanosuspension in *Terminalia bellirica* fruit aqueous extract for catalytic and antibacterial applications. 3 Biotech..

[66] Naraginti S., Li Y. (2017). Preliminary investigation of catalytic, antioxidant, anticancer and bactericidal activity of green synthesized silver and gold nanoparticles using *Actinidia deliciosa*. J. Photochem. Photobiol. B..

[67] Aboelfetoh E.F., El-Shenody R.A., Ghobara M.M. (2017). Eco-friendly synthesis of silver nanoparticles using green algae (*Caulerpa serrulata*): Reaction optimization, catalytic and antibacterial activities. Environ. Monit. Assess..

[68] Bonigala B., Mangamuri U.K., Anuhya G., Yamini Saraswathi Y., Rao K.R.S.S., Poda S. (2017). Green Synthesis of Silver Nanoparticles using two *Apocyanaceae* plants and Screening for their Catalytic activity. Curr. Trends Biotechnol. Pharm..

[69] Baghbani-Arani F., Movagharnia R., Sharifian A., Salehi S., Shandiz S.A.S. (2017). Photo-catalytic, anti-bacterial, and anti-cancer properties of phyto-mediated synthesis of silver nanoparticles from *Artemisia tournefortiana* Rchb extract. J. Photochem. Photobiol. B..

[70] Sapkota K., Narayanan K.B., Han S.S. (2017). Environmentally Sustainable Synthesis of Catalytically-Active Silver Nanoparticles and Their Cytotoxic Effect on Human Keratinocytes. J. Clust. Sci..

[71] Issaabadi Z., Nasrollahzadeh M., Sajadi S.M. (2017). Efficient catalytic hydration of cyanamides in aqueous medium and in the presence of Naringin sulfuric acid or green synthesized silver nanoparticles by using *Gongronema latifolium* leaf extract. J. Colloid Interface Sci..

[72] Balwe S.G., Shinde V.V., Rokade A.A., Park S.S., Jeong Y.T. (2017). Green synthesis and characterization of silver nanoparticles (Ag NPs) from extract of plant *Radix Puerariae*: An efficient and recyclable catalyst for the construction of pyrimido[1,2-*b*]indazole derivatives under solvent-free conditions. Catal. Commun..

[73] Zaheer Z. (2018). Biogenic synthesis, optical, catalytic, and in vitro antimicrobial potential of Ag-nanoparticles prepared using Palm date fruit extract. J. Photochem. Photobiol. B..

[74] Arya G., Kumari R.M., Gupta N., Kumar A., Chandra R., Nimesh S. (2018). Green synthesis of silver nanoparticles using *Prosopis juliflora* bark extract: Reaction optimization, antimicrobial and catalytic activities. Artif. Cells Nanomed. Biotechnol..

[75] Vijayan R., Joseph S., Mathew B. (2018). *Indigofera tinctoria* leaf extract mediated green synthesis of silver and gold nanoparticles and assessment of their anticancer, antimicrobial, antioxidant and catalytic properties. Artif. Cells Nanomed. Biotechnol..

[76] Vijayan R., Joseph S., Mathew B. (2018). Augmented antimicrobial, antioxidant and catalytic activities of green synthesised silver nanoparticles. Mater. Res. Express.

[77] Francis S., Joseph S., Koshy E.P., Mathew B. (2018). Microwave assisted green synthesis of silver nanoparticles using leaf extract of *Elephantopus scaber* and its environmental and biological applications. Artif. Cells Nanomed. Biotechnol..

[78] Bonigala B., Kasukurthi B., Konduri V.V., Mangamuri U.K., Gorrepati R., Poda S. (2018). Green synthesis of silver and gold nanoparticles using *Stemona tuberosa* Lour and screening for their catalytic activity in the degradation of toxic chemicals. Environ. Sci. Pollut. Res. Int..

[79] Vijayan R., Joseph S., Mathew B. (2018). Green Synthesis, Characterization and Applications of Noble Metal Nanoparticles Using *Myxopyrum serratulum* A.W. Hill Leaf Extract. Bio. Nano Sci..

[80] Khan A.U., Yuan Q., Khan Z.U.H., Ahmad A., Khan F.U., Tahir K., Shakeel M., Ullah S. (2018). An eco-benign synthesis of AgNPs using aqueous extract of Longan fruit peel: Antiproliferative response against human breast cancer cell line MCF-7, antioxidant and photocatalytic deprivation of methylene blue. J. Photochem. Photobiol. B..

[81] Sumitha S., Vasanthi S., Shalini S., Chinni S.V., Gopinath S.C.B., Anbu P., Bahari M.B., Harish R., Kathiresan S., Ravichandran V. (2018). Phyto-Mediated Photo Catalysed Green Synthesis of Silver Nanoparticles Using *Durio Zibethinus* Seed Extract: Antimicrobial and Cytotoxic Activity and Photocatalytic Applications. Molecules.

[82] Rasheed T., Bilal M., Li C., Nabeel F., Khalid M., Iqbal H.M.N. (2018). Catalytic potential of bio-synthesized silver nanoparticles using *Convolvulus arvensis* extract for the degradation of environmental pollutants. J. Photochem. Photobiol. B..

[83] Saravanakumar K., Chelliah R., Shanmugam S., Varukattu N.B., Oh D.H., Kathiresan K., Wang M.H. (2018). Green synthesis and characterization of biologically active nanosilver from seed extract of *Gardenia jasminoides* Ellis. J. Photochem. Photobiol. B..

[84] Sharma P., Pant S., Rai S., Yadav R.B., Dave V. (2018). Green Synthesis of Silver Nanoparticle Capped with *Allium cepa* and Their Catalytic Reduction of Textile Dyes: An Ecofriendly Approach. J. Polym. Environ..

[85] Vijayan R., Joseph S., Mathew B. (2018). Green Synthesis, Green synthesis of silver nanoparticles using *Nervalia zeylanica* leaf extract and evaluation of their antioxidant, catalytic, and antimicrobial potentials. Particul. Sci. Technol..

[86] Ramar K., Vasanthakumar V., Priyadharsan A., Priya P., Raj V., Anbarasan P.M., Vasanthakumari R., Jafar Ahamed A. (2018). Green synthetic approach of silver nanoparticles from *Bauhinia tomentosa* Linn. leaves extract for potent photocatalytic and in vitro biological applications. J. Mater. Sci. Mater. El..

[87] Nakkala J.R., Mata R., Raja K., Khub Chandra V., Sadras S.R. (2018). Green synthesized silver nanoparticles: Catalytic dye degradation, in vitro anticancer activity and in vivo toxicity in rats. Mater. Sci. Eng. C Mater. Biol. Appl..

[88] Wang F., Zhang W., Tan X., Wang Z., Li Y., Li W. (2019). Extract of *Ginkgo biloba* leaves mediated biosynthesis of catalytically active and recyclable silver nanoparticles. Colloid Surface. A.

[89] Vijayan R., Joseph S., Mathew B. (2019). Anticancer, antimicrobial, antioxidant, and catalytic activities of green-synthesized silver and gold nanoparticles using *Bauhinia purpurea* leaf extract. Bioprocess. Biosyst. Eng..

[90] Yu C., Tang J., Liu X., Ren X., Zhen M., Wang L. (2019). Green Biosynthesis of Silver Nanoparticles Using *Eriobotrya japonica* (Thunb.) Leaf Extract for Reductive Catalysis. Materials.

[91] Gupta N., Singh H.P., Sharma R.K. (2010). Single-pot synthesis: Plant mediated gold nanoparticles catalyzed reduction of methylene blue in presence of stannous chloride. Colloid Surf. A.

[92] Ghosh S., Patil S., Ahire M., Kitture R., Gurav D.D., Jabgunde A.M., Kale S., Pardesi K., Shinde V., Bellare J. (2012). *Gnidia glauca* flower extract mediated synthesis of gold nanoparticles and evaluation of its chemocatalytic potential. J. Nanobiotechnol..

[93] Zayed M.F., Eisa W.H. (2014). Phoenix dactylifera L. leaf extract phytosynthesized gold nanoparticles; controlled synthesis and catalytic activity. Spectrochim. Acta A Mol. Biomol. Spectrosc..

[94] Ayaz Ahmed K.B., Subramanian S., Sivasubramanian A., Veerappan G., Veerappan A. (2014). Preparation of gold nanoparticles using *Salicornia brachiata* plant extract and evaluation of catalytic and antibacterial activity. Spectrochim. Acta A Mol. Biomol. Spectrosc..

[95] Borhamdin S., Shamsuddin M., Alizadeh A. (2016). Biostabilised icosahedral gold nanoparticles: Synthesis, cyclic voltammetric studies and catalytic activity towards 4-nitrophenol reduction. J. Exp. Nanosci..

[96] Ghosh S., Patil S., Chopade N.B., Luikham S., Kitture R., Gurav D.D., Patil A.B., Phadatare S.D., Sontakke V., Kale S. (2016). *Gnidia glauca* Leaf and Stem Extract Mediated Synthesis of Gold Nanocatalysts with Free Radical Scavenging Potential. J. Nanomed. Nanotechnol..

[97] Lim S.H., Ahn E.Y., Park Y. (2016). Green Synthesis and Catalytic Activity of Gold Nanoparticles Synthesized by *Artemisia capillaris* Water Extract. Nanoscale Res. Lett..

[98] Dauthal P., Mukhopadhyay M. (2016). Phyto-synthesis and structural characterization of catalytically active gold nanoparticles biosynthesized using *Delonix regia* leaf extract. 3 Biotech.

[99] Choudhary B.C., Paul D., Gupta T., Tetgure S.R., Garole V.J., Borse A.U., Garole D. (2016). Photocatalytic reduction of organic pollutant under visible light by green route synthesized gold nanoparticles. J. Environ. Sci..

[100] Khan S., Runguo W., Tahir K., Jichuan Z., Zhang L. (2017). Catalytic reduction of 4-nitrophenol and photo inhibition of *Pseudomonas aeruginosa* using gold nanoparticles as photocatalyst. J. Photochem. Photobiol. B..

[101] Khan Z.U.H., Khan A., Chen Y., Khan A.U., Shah N.S., Muhammad N., Tahir B.M.K., Khan F.U., Pingyu W. (2017). Photo catalytic applications of gold nanoparticles synthesized by green route and electrochemical degradation of phenolic Azo dyes using AuNPs /GC as modified paste electrode. J. Alloy. Compd..

[102] Wacławek S., Gončuková Z., Adach K., Fijałkowski M., Černík M. (2018). Green synthesis of gold nanoparticles using *Artemisia dracunculus* extract: Control of the shape and size by varying synthesis conditions. Environ. Sci. Pollut. Res. Int..

[103] Teimouri M., Khosravinejad F., Attar F., Saboury A.A., Kostova I., Benelli G., Falahati M. (2018). Gold nanoparticles fabrication by plant extracts: Synthesis, characterization, degradation of 4-nitrophenol from industrial wastewater, and insecticidal activity—A review. J. Clean. Prod..

[104] Matthews R.D., Bottomley L.A., Pavlostathis S.G. (2009). Palladium-catalyzed hydrogen reduction and decolorization of reactive phthalocyanine dyes. Desalination.

[105] Bhat I.U.H., Anwar M.N.K., Appaturi J.N. (2019). Polymer Based Palladium Nanocatalyst for the Degradation of Nitrate and Congo Red. J. Polym. Environ..

[106] Khan M., Khan M., Kuniyil M., Adil S.F., Al-Warthan A., Alkhathlan H.Z., Tremel W., Tahir M.N., Siddiqui M.R. (2014). Biogenic synthesis of palladium nanoparticles using *Pulicaria glutinosa* extract and their catalytic activity towards the Suzuki coupling reaction. Dalton Trans..

[107] Veisi H., Ghorbani-Vaghei R., Hemmati S., Aliani M.H., Ozturk T. (2015). Green and effective route for the synthesis of monodispersed palladium nanoparticles using herbal tea extract (*Stachys lavandulifolia*) as reductant, stabilizer and capping agent, and their application as homogeneous and reusable catalyst in Suzuki coupling reactions in water. Appl. Organomet. Chem..

[108] Nasrollahzadeh M., Sajadi S.M., Honarmand E., Maham M. (2015). Preparation of palladium nanoparticles using *Euphorbia thymifolia* L. leaf extract and evaluation of catalytic activity in the ligand-free Stille and Hiyama cross-coupling reactions in water. New J. Chem..

[109] Duan L., Li M., Liu H. (2015). Biosynthesised palladium nanoparticles using *Eucommia ulmoides* bark aqueous extract and their catalytic activity. IET Nanobiotechnol..

[110] Nasrollahzadeh M., Mohammad Sajadi S. (2016). Pd nanoparticles synthesized in situ with the use of *Euphorbia granulate* leaf extract: Catalytic properties of the resulting particles. J. Colloid Interface Sci..

[111] Tahir K., Nazir S., Li B., Ahmad A., Nasir T., Khan A.U., Shah S.A., Khan Z.U., Yasin G., Hameed M.U. (2016). *Sapium sebiferum* leaf extract mediated synthesis of palladium nanoparticles and in vitro investigation of their bacterial and photocatalytic activities. J. Photochem. Photobiol. B.

[112] Hazarika M., Borah D., Bora P., Silva A.R., Das P. (2017). Biogenic synthesis of palladium nanoparticles and their applications as catalyst and antimicrobial agent. PLoS ONE.

[113] Sharmila G., Haries S., Farzana Fathima M., Geetha S., Manoj Kumar N., Muthukumaran C. (2017). Enhanced catalytic and antibacterial activities of phytosynthesized palladium nanoparticles using *Santalum album* leaf extract. Powder Technol..

[114] Lebaschi S., Hekmati M., Veisi H. (2017). Green synthesis of palladium nanoparticles mediated by black tea leaves (*Camellia sinensis*) extract: Catalytic activity in the reduction of 4-nitrophenol and Suzuki-Miyaura coupling reaction under ligand-free conditions. J. Colloid Interface Sci..

[115] Narasaiah P., Mandal B.K., Sarada N.C. (2017). Green synthesis of Pd NPs from *Pimpinella tirupatiensis* plant extract and their application in photocatalytic activity dye degradation. IOP Conf. Ser. Mater. Sci. Eng..

[116] Shaik M.R., Ali Z.J., Khan M., Kuniyil M., Assal M.E., Alkhathlan H.Z., Al-Warthan A., Siddiqui M.R., Khan M., Adil S.F. (2017). Green Synthesis and Characterization of Palladium Nanoparticles Using Origanum vulgare L. Extract and Their Catalytic Activity. Molecules.

[117] Garole V.J., Choudhary B.C., Tetgure S.R., Garole D.J., Borse A.U. (2019). Palladium nanocatalyst: Green synthesis, characterization, and catalytic application. J. Environ. Sci. Technol..

[118] Njagi E.C., Huang H., Stafford L., Genuino H., Galindo H.M., Collins J.B., Hoag G.E., Suib S.L. (2011). Biosynthesis of Iron and Silver Nanoparticles at Room Temperature Using Aqueous Sorghum Bran Extracts. Langmuir.

[119] Machado S., Stawiński W., Slonina P., Pinto A.R., Grosso J.P., Nouws H.P., Albergaria J.T., Delerue-Matos C. (2013). Application of green zero-valent iron nanoparticles to the remediation of soils contaminated with ibuprofen. Sci. Total. Environ..

[120] Lin J., Weng X., Dharmarajan R., Chen Z. (2017). Characterization and reactivity of iron based nanoparticles synthesized by tea extracts under various atmospheres. Chemosphere.

[121] Garole V.J., Choudhary B.C., Tetgure S.R., Garole D.J., Borse A.U. (2018). Detoxification of toxic dyes using biosynthesized iron nanoparticles by photo-Fenton processes. Int. J. Environ. Sci. Technol..

[122] Radini I.A., Hasan N., Malik M.A., Khan Z. (2018). Biosynthesis of iron nanoparticles using *Trigonella foenum-graecum* seed extract for photocatalytic methyl orange dye degradation and antibacterial applications. J. Photochem. Photobiol. B.

[123] Khan Z., Al-Thabaiti S.A. (2018). Green synthesis of zero-valent Fe-nanoparticles: Catalytic degradation of rhodamine B, interactions with bovine serum albumin and their enhanced antimicrobial activities. J. Photochem. Photobiol. B.

[124] Nasrollahzadeh M., Sajadi S.M. (2015). Green synthesis of copper nanoparticles using *Ginkgo biloba* L. leaf extract and their catalytic activity for the Huisgen [3+2] cycloaddition of azides and alkynes at room temperature. J. Colloid Interface Sci..

[125] Prasad P.R., Kanchi S., Naidoo E.B. (2016). In-vitro evaluation of copper nanoparticles cytotoxicity on prostate cancer cell lines and their antioxidant, sensing and catalytic activity: One-pot green approach. J. Photochem. Photobiol. B.

[126] Nazar N., Bibi I., Kamal S., Iqbal M., Nouren S., Jilani K., Umair M., Ata S. (2018). Cu nanoparticles synthesis using biological molecule of P. granatum seeds extract as reducing and capping agent: Growth mechanism and photo-catalytic activity. Int. J. Biol. Macromol..

[127] Nasrollahzadeh M., Momeni S.S., Sajadi S.M. (2017). Green synthesis of copper nanoparticles using *Plantago asiatica* leaf extract and their application for the cyanation of aldehydes using K_4_Fe(CN)_6_. J. Colloid Interface Sci..

[128] Ravi G., Loganathan B., Karthikeyan R. (2015). Green Synthesis of Au-Ag Bimetallic Nanocomposite using *Silybum marianum* Seed Extract and Their Application as Catalyst. RSC Adv..

[129] Luo F., Yang D., Chen Z., Megharaj M., Naidu R. (2016). One-step green synthesis of bimetallic Fe/Pd nanoparticles used to degrade Orange II. J. Hazard. Mater..

[130] Al-Asfar A., Zaheer Z., Aazam E.S. (2018). Eco-friendly green synthesis of Ag@Fe bimetallic nanoparticles: Antioxidant, antimicrobial and photocatalytic degradation of bromothymol blue. J. Photochem. Photobiol. B.

[131] Taghizadeh S.M., Berenjian A., Taghizadeh S., Ghasemi Y., Taherpour A., Sarmah A.K., Ebrahiminezha A. (2019). One-put green synthesis of multifunctional silver iron core-shell nanostructure with antimicrobial and catalytic properties. Ind. Crop. Prod..

[132] Sharma R.K., Dutta S., Sharma S., Zboril R., Varma R.S., Gawande M.B. (2016). Fe_3_O_4_ (iron oxide)- supported nanocatalysts: Synthesis, characterization and applications in coupling reactions. Green Chem..

[133] Singh J., Dutta T., Kim K.H., Rawat M., Samddar P., Kumar P. (2018). ‘Green’ synthesis of metals and their oxide nanoparticles: Applications for environmental remediation. J. Nanobiotechnol..

[134] Basnet P., Inakhunbi Chanu T., Samanta D., Chatterjee S. (2018). A review on bio-synthesized zinc oxide nanoparticles using plant extracts as reductants and stabilizing agents. J. Photochem. Photobiol. B.

[135] Basavegowda N., Magar K.B.S., Mishra K., Lee Y.R. (2014). Green fabrication of ferromagnetic Fe_3_O_4_ nanoparticles and their novel catalytic applications for the synthesis of biologically interesting benzoxazinone and benzthioxazinone derivatives. New J. Chem..

[136] Vasantharaj S., Sathiyavimal S., Senthilkumar P., Lewis Oscar F., Pugazhendhi A. (2019). Biosynthesis of iron oxide nanoparticles using leaf extract of *Ruellia tuberosa*: Antimicrobial properties and their applications in photocatalytic degradation. J. Photochem. Photobiol. B.

[137] Nasrollahzadeh M., Maham M., Sajadi S.M. (2015). Green synthesis of CuO nanoparticles by aqueous extract of *Gundelia tournefortii* and evaluation of their catalytic activity for the synthesis of N-monosubstituted ureas and reduction of 4-nitrophenol. J. Colloid Interface Sci..

[138] Nasrollahzadeh M., Mohammad Sajadi S., Rostami-Vartooni A., Hussin S.M. (2016). Green synthesis of CuO nanoparticles using aqueous extract of *Thymus vulgaris* L. leaves and their catalytic performance for N-arylation of indoles and amines. J. Colloid Interface Sci..

[139] Devi H.S., Singh T.D., Singh H.P. (2017). Optically understanding the dependence of catalysis kinetics on work function of nanocatalyst. Bull. Mater. Sci..

[140] Sathiyavimal S., Vasantharaj S., Bharathi D., Saravanan M., Manikandan E., Kumar S.S., Pugazhendhi A. (2018). Biogenesis of copper oxide nanoparticles (CuONPs) using *Sida acuta* and their incorporation over cotton fabrics to prevent the pathogenicity of Gram negative and Gram positive bacteria. J. Photochem. Photobiol. B.

[141] Prakash S., Elavarasan N., Venkatesan A., Subashini K., Sowndharya M., Sujatha V. (2018). Green synthesis of copper oxide nanoparticles and its effective applications in Biginelli reaction, BTB photodegradation and antibacterial activity. Adv. Powder Technol..

[142] Gu H., Chen X., Chen F., Zhou X., Parsaee Z. (2018). Ultrasound-assisted biosynthesis of CuO-NPs using brown alga *Cystoseira trinodis*: Characterization, photocatalytic AOP, DPPH scavenging and antibacterial investigations. Ultrason. Sonochem..

[143] Suresh D., Shobharani R.M., Nethravathi P.C., Pavan Kumar M.A., Nagabhushana H., Sharma S.C. (2015). *Artocarpus gomezianus* aided green synthesis of ZnO nanoparticles: Luminescence, photocatalytic and antioxidant properties. Spectrochim. Acta A Mol. Biomol. Spectrosc..

[144] Anbuvannan M., Ramesh M., Viruthagiri G., Shanmugam N., Kannadasan N. (2015). Synthesis, characterization and photocatalytic activity of ZnO nanoparticles prepared by biological method. Spectrochim. Acta A Mol. Biomol. Spectrosc..

[145] Patel V.K., Sundriyal P., Bhattacharya S. (2017). *Aloe vera* vs. poly(ethylene)glycol-based synthesis and relative catalytic activity investigations of ZnO nanorods in thermal decomposition of potassium perchlorate. Particul. Sci. Technol..

[146] Raja A., Ashokkumar S., Pavithra Marthandam R., Jayachandiran J., Khatiwda C.P., Kaviyarasu K., Ganapathi Raman R., Swaminathan M. (2018). Eco-friendly preparation of zinc oxide nanoparticles using *Tabernaemontana divaricata* and its photocatalytic and antimicrobial activity. J. Photochem. Photobiol. B.

[147] Ishwarya R., Vaseeharan B., Kalyani S., Banumathi B., Govindarajan M., Alharbi N.S., Kadaikunnan S., Al-Anbr M.N., Khaled J.M., Benelli G. (2018). Facile green synthesis of zinc oxide nanoparticles using *Ulva lactuca* seaweed extract and evaluation of their photocatalytic, antibiofilm and insecticidal activity. J. Photochem. Photobiol. B.

[148] Ali J., Irshad R., Li B., Tahir K., Ahmad A., Shakeel M., Khan N.U., Khan Z.U.H. (2018). Synthesis and characterization of phytochemical fabricated zinc oxide nanoparticles with enhanced antibacterial and catalytic applications. J. Photochem. Photobiol. B.

[149] Phukan S., Mahanta A., Rashid M.H. (2018). Size-tunable ZnO nanotapes as an efficient catalyst for oxidative chemoselective C-B bond cleavage of arylboronic acids. Appl.Catal. A.

[150] Khan Z.U.H., Sadiq H.M., Shah N.S., Khan A.U., Muhammad N., Hassan S.U., Tahir K., Shafi S.Z., Khan F.U., Imran M. (2019). Greener synthesis of zinc oxide nanoparticles using *Trianthema portulacastrum* extract and evaluation of its photocatalytic and biological applications. J. Photochem. Photobiol. B.

[151] Haritha E., Roopan S.M., Madhavi G., Elango G., Al-Dhabi N.A., Arasu M.V. (2016). Green chemical approach towards the synthesis of SnO_2_ NPs in argument with photocatalytic degradation of diazo dye and its kinetic studies. J. Photochem. Photobiol. B.

[152] Begum S., Ahmaruzzaman M. (2018). Green synthesis of SnO_2_ quantum dots using *Parkia speciosa* Hassk pods extract for the evaluation of anti-oxidant and photocatalytic properties. J. Photochem. Photobiol. B.

[153] Thandapani K., Kathiravan M., Namasivayam E., Padiksan I.A., Natesan G., Tiwari M., Giovanni B., Perumal V. (2018). Enhanced larvicidal, antibacterial, and photocatalytic efficacy of TiO_2_ nanohybrids green synthesized using the aqueous leaf extract of *Parthenium hysterophorus*. Environ. Sci. Pollut. Res. Int..

[154] Udayabhanu J., Kannan V., Tiwari M., Natesan G., Giovanni B., Perumal V. (2018). Nanotitania crystals induced efficient photocatalytic color degradation, antimicrobial and larvicidal activity. J. Photochem. Photobiol. B.

[155] Sharma J.K., Srivastava P., Ameen S., Akhtar M.S., Singh G., Yadava S. (2016). *Azadirachta indica* plant-assisted green synthesis of Mn_3_O_4_ nanoparticles: Excellent thermal catalytic performance and chemical sensing behavior. J. Colloid. Interface Sci..

[156] Surendra T.V., Roopan S.M. (2016). Photocatalytic and antibacterial properties of phytosynthesized CeO_2_ NPs using *Moringa oleifera* peel extract. J. Photochem. Photobiol. B.

[157] Sai Saraswathi V., Santhakumar K. (2017). Photocatalytic activity against azo dye and cytotoxicity on MCF-7 cell lines of zirconium oxide nanoparticle mediated using leaves of *Lagerstroemia speciosa*. J. Photochem. Photobiol. B.

[158] Ezhilarasi A.A., Vijaya J.J., Kaviyarasu K., Kennedy L.J., Jothiramalingam R., Al-Lohedan H.A. (2018). Green synthesis of NiO nanoparticles using *Aegle marmelos* leaf extract for the evaluation of in-vitro cytotoxicity, antibacterial and photocatalytic properties. J. Photochem. Photobiol. B.

[159] Huo Y., Singh P., Kim Y.J., Soshnikova V., Kang J., Markus J., Ahn S., Castro-Aceituno V., Mathiyalagan R., Chokkalingam M. (2018). Biological synthesis of gold and silver chloride nanoparticles by *Glycyrrhiza uralensis* and in vitro applications. Artif. Cells Nanomed. Biotechnol..

[160] Nasrollahzadeh M., Sajadi S.M., Rostami-Vartooni A., Bagherzadeh M. (2015). Green synthesis of Pd/CuO nanoparticles by *Theobroma cacao* L. seeds extract and their catalytic performance for the reduction of 4-nitrophenol and phosphine-free Heck coupling reaction under aerobic conditions. J. Colloid Interface Sci..

[161] Sajadi S.M., Nasrollahzadeh M., Maham M. (2016). Aqueous extract from seeds of *Silybum marianum* L. as a green material for preparation of the Cu/Fe_3_O_4_ nanoparticles: A magnetically recoverable and reusable catalyst for the reduction of nitroarenes. J. Colloid Interface Sci..

[162] Nasrollahzadeh M., Sajadi S.M. (2016). Green synthesis, characterization and catalytic activity of the Pd/TiO_2_ nanoparticles for the ligand-free Suzuki-Miyaura coupling reaction. J. Colloid Interface Sci..

[163] Atarod M., Nasrollahzadeh M., Mohammad Sajadi S. (2016). *Euphorbia heterophylla* leaf extract mediated green synthesis of Ag/TiO_2_ nanocomposite and investigation of its excellent catalytic activity for reduction of variety of dyes in water. J. Colloid Interface Sci..

[164] Momeni S.S., Nasrollahzadeh M., Rustaiyan A. (2016). Green synthesis of the Cu/ZnO nanoparticles mediated by *Euphorbia prolifera* leaf extract and investigation of their catalytic activity. J. Colloid Interface Sci..

[165] Sajjadi M., Nasrollahzadeh M., Mohammad Sajadi S. (2017). Green synthesis of Ag/Fe_3_O_4_ nanocomposite using *Euphorbia peplus* Linn leaf extract and evaluation of its catalytic activity. J. Colloid Interface Sci..

[166] Haq A., Saeed M., Jamal M.A., Akram N., Bokhari T.H., Afaq U. (2018). Green Synthesis of Ag–NiO and Investigation of its Catalytic Activity for Degradation of Rhodamine B Dye in Aqueous Medium. Z. Phys. Chem..

[167] Devi T.B., Ahmaruzzaman M., Begum S. (2016). A rapid, facile and green synthesis of Ag@AgCl nanoparticles for the effective reduction of 2,4-dinitrophenyl hydrazine. New J. Chem..

[168] Devi T.B., Ahmaruzzaman M. (2016). Bio-inspired sustainable and green synthesis of plasmonic Ag/AgCl nanoparticles for enhanced degradation of organic compound from aqueous phase. Environ. Sci. Pollut. Res. Int..

[169] Bharati R., Suresh S. (2016). Synthesis of green ZnO/SiO_2_ nanocatalyst and its application to reduce acenaphthylene from refinery waste water. Biosci. Biotech. Res. Comm..

[170] Maria Magdalane C., Kaviyarasu K., Judith Vijaya J., Jayakumar C., Maaza M., Jeyaraj B. (2017). Photocatalytic degradation effect of malachite green and catalytic hydrogenation by UV-illuminated CeO_2_/CdO multilayered nanoplatelet arrays: Investigation of antifungal and antimicrobial activities. J. Photochem. Photobiol. B.

[171] Kombaiah K., Vijaya K.J., Kennedy L.J., Bououdina M., Al-Lohedan H.A., Ramalingam R.J. (2017). Studies on *Opuntia dilenii haw* mediated multifunctional ZnFe_2_O_4_ nanoparticles: Optical, magnetic and catalytic applications. Mater. Chem. Phys..

[172] Garg S., Yadav M., Chandra A., Gahlawat S., Ingole P.P., Pap Z., Hernadi K. (2018). Plant leaf extracts as photocatalytic activity tailoring agents for BiOCl towards environmental remediation. Ecotoxicol. Environ. Saf..

[173] Zhan G., Du M., Huang J., Li Q. (2011). Green synthesis of Au/TS-1 catalysts via two novel modes and their surprising performance for propylene epoxidation. Catal. Commun..

[174] Nasrollahzadeh M., Sajadi S.M., Hatamifard A. (2015). *Anthemis xylopoda* flowers aqueous extract assisted in situ green synthesis of Cu nanoparticles supported on natural Natrolite zeolite for N-formylation of amines at room temperature under environmentally benign reaction conditions. J. Colloid Interface Sci..

[175] Hatamifard A., Nasrollahzadeh M., Sajadi S.M. (2016). Biosynthesis, characterization and catalytic activity of an Ag/zeolite nanocomposite for base and ligand-free oxidative hydroxylation of phenylboronic acid and reduction of a variety of dyes at room temperature. New J. Chem..

[176] Das R., Ghosh S., Chowdhury I.H., Naskar M.K. (2016). Biogenic silver nanoparticles impregnated hollow mesoporous silicalite-1: An efficient catalyst for p-nitrophenol reduction. New J. Chem..

[177] Nasrollahzadeh M., Sajadi S.M., Rostami-Vartooni A., Alizadeh M., Bagherzadeh M. (2016). Green synthesis of the Pd nanoparticles supported on reduced graphene oxide using barberry fruit extract and its application as a recyclable and heterogeneous catalyst for the reduction of nitroarenes. J. Colloid. Interface Sci..

[178] Atarod M., Nasrollahzadeh M., Sajadi S.M. (2016). Green synthesis of Pd/RGO/Fe3O4 nanocomposite using *Withania coagulans* leaf extract and its application as magnetically separable and reusable catalyst for the reduction of 4-nitrophenol. J. Colloid Interface Sci..

[179] Maham M., Nasrollahzadeh M., Sajadi S.M., Nekoei M. (2017). Biosynthesis of Ag/reduced graphene oxide/Fe_3_O_4_ using *Lotus garcinii* leaf extract and its application as a recyclable nanocatalyst for the reduction of 4-nitrophenol and organic dyes. J. Colloid Interface Sci..

[180] Nasrollahzadeh M., Atarod M., Sajadi S.M. (2017). Biosynthesis, characterization and catalytic activity of Cu/RGO/Fe_3_O_4_ for direct cyanation of aldehydes with K_4_[Fe(CN)_6_]. J. Colloid Interface Sci..

[181] Khan M., Kuniyil M., Shaik M.R., Khan M., Adil S.F., Al-Warthan A., Alkhathlan H.Z., Tremel W., Tahir M.N., Siddiqui H.R.H. (2017). Plant Extract Mediated Eco-Friendly Synthesis of Pd@Graphene Nanocatalyst: An Efficient and Reusable Catalyst for the Suzuki-Miyaura Coupling. Catalysts.

[182] Anasdass J.R., Kannaiyan P., Raghavachary R., Gopinath S.C.B., Chen Y. (2018). Palladium nanoparticle-decorated reduced graphene oxide sheets synthesized using *Ficus carica* fruit extract: A catalyst for Suzuki cross-coupling reactions. PLoS ONE.

[183] Smuleac V., Varma R., Sikdar S., Bhattacharyya D. (2011). Green synthesis of Fe and Fe/Pd bimetallic nanoparticles in membranes for reductive degradation of chlorinated organics. J. Membrane Sci..

[184] Momeni S.S., Nasrollahzadeh M., Rustaiyan A. (2017). Biosynthesis and application of Ag/bone nanocomposite for the hydration of cyanamides in *Myrica gale* L. extract as a green solvent. J. Colloid Interface Sci..

[185] Goswami M., Baruah D., Das A.M. (2018). Green synthesis of silver nanoparticles supported on cellulose and their catalytic application in the scavenging of organic dyes. New J. Chem..

[186] Naeimi A., Honarmand M., Sedri  A. (2019). Ultrasonic assisted fabrication of first MoO_3_/copper complex bio-nanocomposite based on *Sesbania sesban* plant for green oxidation of alcohols. Ultrason. Sonochem..

[187] Rostami-Vartooni A. (2017). Green synthesis of CuO nanoparticles loaded on the seashell surface using *Rumex crispus* seeds extract and its catalytic applications for reduction of dyes. IET Nanobiotechnol..

[188] Bordbar M., Mortazavimanesh N. (2017). Green synthesis of Pd/walnut shell nanocomposite using *Equisetum arvense* L. leaf extract and its application for the reduction of 4-nitrophenol and organic dyes in a very short time. Environ. Sci. Pollut. Res. Int..

[189] Khodadadi B., Bordbar M., Nasrollahzadeh M. (2017). Green synthesis of Pd nanoparticles at Apricot kernel shell substrate using *Salvia hydrangea* extract: Catalytic activity for reduction of organic dyes. J. Colloid Interface Sci..

[190] Khodadadi B., Bordbar M., Nasrollahzadeh M. (2017). *Achillea millefolium* L. extract mediated green synthesis of waste peach kernel shell supported silver nanoparticles: Application of the nanoparticles for catalytic reduction of a variety of dyes in water. J. Colloid Interface Sci..

